# Forward–Backward-Flushing Valve-Assisted Selectivity Tuning (FBF-VAST) for LC × LC: Principles and Demonstration of a Modulation Mechanism

**DOI:** 10.1021/acs.analchem.5c08280

**Published:** 2026-05-11

**Authors:** Pattraporn Chobpradit, Kenji Hamase, Sornkanok Vimolmangkang, Thumnoon Nhujak, Chadin Kulsing

**Affiliations:** † Department of Chemistry, Faculty of Science, 133942 Chulalongkorn University, Bangkok 10330, Thailand; ‡ Graduate School of Pharmaceutical Sciences, 12923 Kyushu University, 3-1-1 Maidashi, Higashi-ku, Fukuoka 812-8582, Japan; § Department of Pharmacognosy and Pharmaceutical Botany, Faculty of Pharmaceutical Sciences, 54772 Chulalongkorn University, Bangkok 10330, Thailand; ∥ Phyto Analytica Testing Laboratory, Leapdelab Company Limited, Samut Prakan 10130, Thailand; ⊥ Center of Excellence in Metabolomics for Life Sciences, Chulalongkorn University, Bangkok 10330, Thailand

## Abstract

A modulation mechanism for comprehensive two-dimensional liquid chromatography (LC × LC), termed FBF-VAST (forward–backward-flushing valve-assisted selectivity tuning), is introduced. This technique enables modulation using a simple system configuration centered around a 4-port valve. The concept lies in periodic forward–backward flushing of the first-dimension (^1^D) column, enabling synchronized analyte holding and release with a stepped-gradient transferred to the second dimension (^2^D). The modulation period (*P*_M_) is defined by the forward- and backward-flushing durations (*t*_fwd_ + *t*_bwd_), with the net injection window (Δ*t*_net_ = *t*_fwd_ – *t*_bwd_) delivering analyte fractions into the ^2^D-column. These processes were simulated using advection–dispersion equations modified to include analyte retention, resulting in solvent and analyte space–time profiles, along with animations that illustrate the modulation mechanism. A unique selectivity-tuning effect was demonstrated through the ^1^D-column simulation and LC × LC experiments using two C18 columns operated under a unified binary gradient for red wine sample separation, yielding 27–55 separated peaks of interest. The selectivity can be effectively adjusted solely by tuning the Valve A timing (*t*_fwd_) or gradient steepness—without altering mobile phase composition or stationary phase chemistry. Repeatability studies showed %RSDs of peak areas within 5.28–11.77% (*n* = 6). In addition, the method enabled backflushing for online ^1^D-column cleaning, which may extend column life and improve robustness. This cost-effective and tunable LC × LC strategy offers enhanced control over chromatographic selectivity and long-term stability, all within a simplified instrumental design.

## Graphical abstract

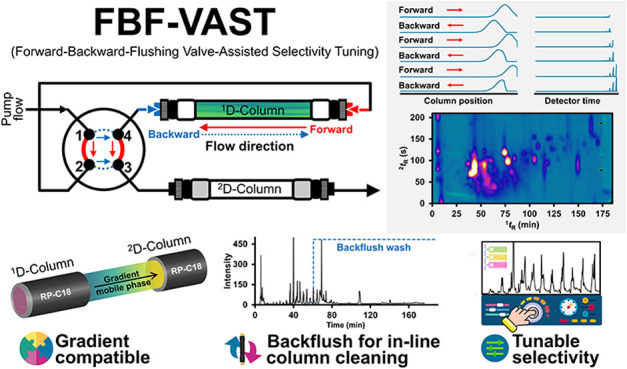

## Introduction

Two-dimensional liquid chromatography (2D-LC) is a powerful platform for resolving complex or impure mixtures in biopharmaceuticals, polymers, foods, and environmental samples.^1–4^ Among its configurations, LC × LC uses two columns connected by a modulator to improve untargeted analysis by increasing the number of resolved peaks.^5–8^ This approach delivers a higher peak capacity per unit time, enabling high-resolution and high-throughput separations.

Passive or nonfocusing modulation remains the predominant strategy in LC × LC, largely due to its operational simplicity.^5,9^ However, this also has some disadvantages such as incompatibility of solvents applied in the first and the second dimensional (^1^D and ^2^D) separations and limitations in peak focusing.^5,10^ In response, active modulation strategies, which address these challenges by enabling solvent compatibility and peak compression, have been developed.^11^ These, however, frequently require two-pump systems, multiport valve setups, auxiliary trapping columns, or specialized cryogenic or membrane-based devices, some of which are not commercially accessible or require complex customization.^12,13^

The backflush technique, although historically rooted in column cleaning applications, presents an underexplored opportunity in this context. Initially introduced in 1976 for removing fat residues at the column head using a strong organic solvent in a 1D-HPLC system,^14^ backflushing has since been employed to protect analytical columns from contamination in both HPLC and GC workflows.^15,16^ More recently, it has been repurposed for sample enrichment in precolumns^17^ and adapted across a variety of platforms including HPLC/GC hyphenation^18^ and inlet backflush-based GC × GC configurations.^19^ The development of a backflush-capable LC × LC system that maintains instrumental simplicity remains a significant technical challenge.

As a case study, red wine provides a highly complex matrix with a substantial commercial relevance. It contains a diverse array of polyphenolic compounds derived from secondary plant metabolism. These constituents contribute significantly to wine’s organoleptic qualities (e.g., flavor, mouthfeel, aroma) and bioactivity.^20^ Due to their broad dynamic range and structural diversity, one-dimensional HPLC techniques often fall short in separating and quantifying these analytes. High-resolution LC × LC methods could address these challenges, provided they are implemented in a cost-effective and operationally simple manner.^21–23^

Here, a new modulation strategy termed forward–backward-flushing valve-assisted selectivity tuning (FBF-VAST) is introduced. The mechanism uses a single 4-port valve to periodically forward- and backward-flush the^1^D-column, thereby controlling the timing and composition of analyte bands introduced into the ^2^D-column. The method exploits the increasing mobile phase strength during flushing events, which introduces an analyte zone reshaping and dynamic enrichment without requiring cryogenic cooling or external trapping cartridges. The heart of the FBF-VAST concept lies in its ability to dynamically tune selectivity using only valve timing and gradient design, even with a single mobile phase system and stationary phase chemistry. This offers an important breakthrough for practical LC × LC implementation, where simplifying hardware without compromising separation power is desired.^24^

The system also supports postseparation backflushing of the ^1^D-column, enabling online column cleaning after each LC × LC run. This feature helps prevent long-term fouling from sample matrices (e.g., proteins or pigments in wine), minimizes carryover, and extends column lifespan—all without requiring additional pumps or switching valves. The dual utility of the same valve configuration for both modulation and maintenance highlights the system’s efficiency, robustness, and practical adaptability for routine multidimensional analysis. To the best of our knowledge, this represents the simplest LC × LC configuration capable of fully gradient-compatible selectivity tuning while retaining backflush functionality.

## Experimental Section

### Simulation of the Results

Simulation of the FBF-VAST modulation was performed using Python, employing advection–dispersion equations modified to include analyte retention along the ^1^D-column. Forward and backward flushing cycles were implemented as time-dependent boundary conditions, allowing the calculation of analyte concentration profiles in both space and time. Further methodological details are provided in the sections Theoretical Background and Simulation of Mobile Phase Gradient Dynamics in FBF-VAST.

### Chemicals and Materials

Dichloromethane (DCM), ethanol, and acetonitrile were sourced from Merck (Darmstadt, Germany) with magnesium sulfate anhydrous from Panreac (Barcelona, Spain). Benzoic acid, caffeine, caftaric acid, (+)-catechin, chlorogenic acid, (−)-epicatechin, gallic acid, nicotinic acid, *p*-coumaric acid, resveratrol, and vanillic acid from Sigma-Aldrich (St. Louis, MO, USA). Wine samples listed in Table S1 were obtained from various locations in Thailand, Australia, and France.

### Sample Preparation

A 10 mL sample of wine was extracted three times with 25.0 mL of DCM to reduce highly polar components such as sugars and anthocyanins, thereby simplifying the matrix and enriching phenolic and other moderately hydrophobic compounds in the extract.^25,26^ The organic extract was dried over anhydrous magnesium sulfate and then filtered.^27^ The solvent was evaporated under N_2_ gas using an automated evaporator (TurboVap Classic LV automated evaporation system, Biotage). Wine extracts of 10.0 mg were dissolved in 1 mL of ethanol and filtrated using filter membranes (W1–W10) before injection into the LC. A crude W4 wine solution was selected for analysis using 1D-LC and 2D-LC to study parameter effects in the developed method.

Each standard stock solution was prepared by dissolving it in suitable solvents such as methanol, ethanol, or water to a concentration of 1000 mg/L.

A 25.0 mL wine sample (W6) was evaporated to dryness. The residue was then reconstituted with 5.0 mL of ethanol and filtered through a membrane filter prior to LC injection. The resulting solution is referred to as the preconcentrated wine (PW6) and was used as an example application in the section dealing with more complex samples.

### LC Instrument

FBF-VAST LC × LC system was developed on an HPLC Agilent 1260 infinity model (Agilent, California, US) consisting of a quaternary pump (G7111B) connected to a vial sampler (G7129A) and a multicolumn thermostat (G7116A) and detected using a diode array detector WR (DAD, G7115A). The 2D system was connected by a controller 4-port dual-position valve (VICI, Schenkon, Switzerland): A position (vertical) and B position (horizon). The reverse-phase columns, including Water AccQ-Tag C18 (3.9 × 150 mm, 4.0 μm) and Agilent ZORBAX Eclipse Plus C18 (4.6 × 100 mm, 3.5 μm) were used as ^1^D and ^2^D-column.

Mobile phase consisted of Milli-Q water (A) and acetonitrile (B) with various gradient mobile phase steps (as gradient mobile phase steps detail in Table S2). The flow rate was set to 0.5 mL/min with an injection volume of 10.0 μL. A UV–vis detector (DAD WR) at a wavelength of 210 nm was applied to detect the separated components in the wine samples. The DAD spectrum scan mode was used to obtain spectrum from each peak. The valve was activated 2 min after injection.

### FBF-VAST LC × LC System Configuration

The LC autoinjector valve was connected to the valve modulator at port 1. The ^1^D-column was installed at ports 2 and 4, while the ^2^D-column was installed at port 3 (Figure 1A). Valve A position refers to the forward flow direction (*t*_fwd_) and Valve B position refers to the backward flow direction (*t*_bwd_).

**Figure 1:**
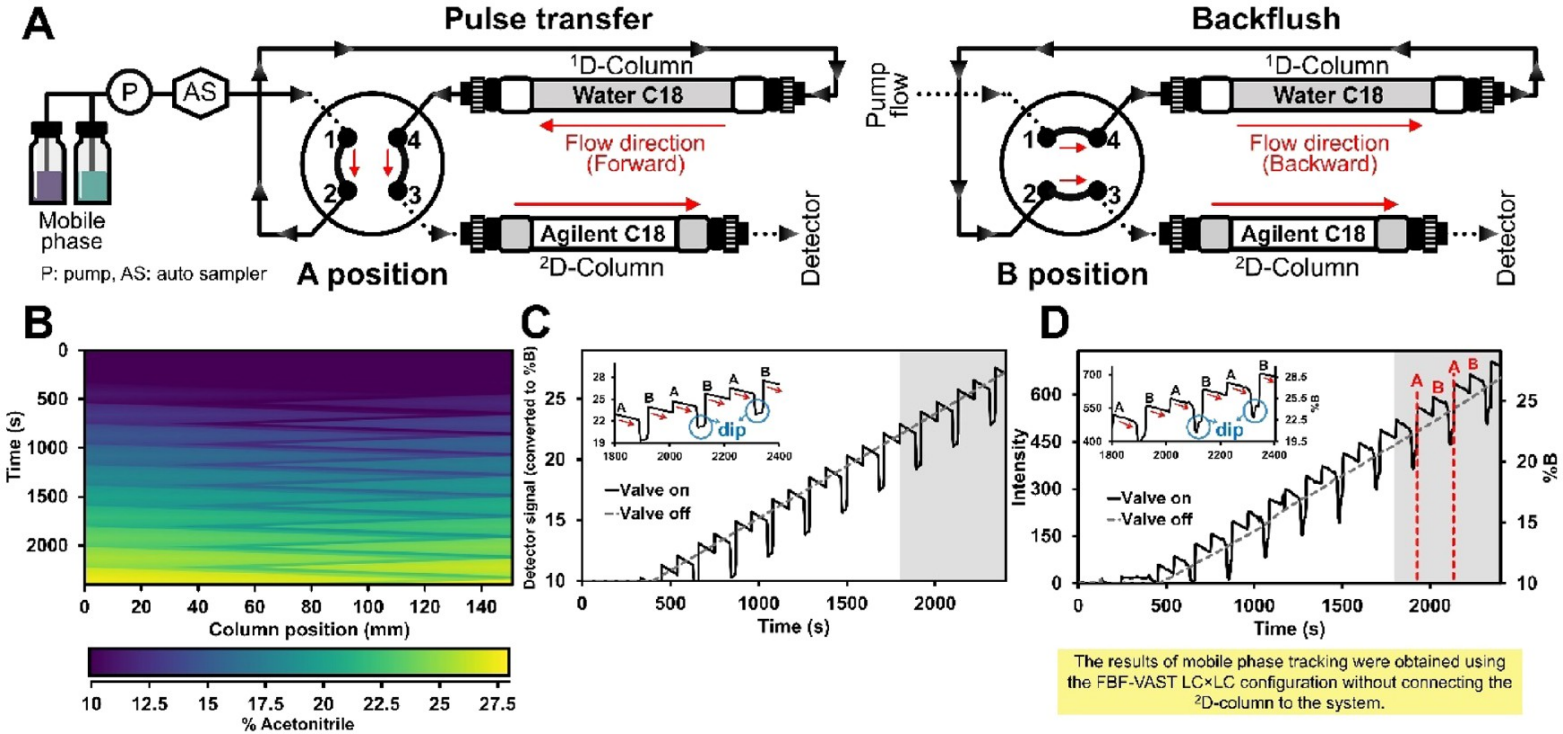
Schematic diagram of the FBF-VAST LC × LC configuration (A), details of %gradient mobile phase behavior during valve switching (B) and the mobile phase tracking graph when the valve switching position of the simulated result for acetone detection (C) together with the experimental result (D).

### Stop-Flow LC × LC System Configuration

The LC autoinjector valve was connected to the valve modulator at port 1. The ^1^D-column was installed at ports 2 and 3, while the ^2^D-column was installed at port 4 (Figure S1). The other parts were the same as those for the FBF-VAST LC × LC system. Valve A position refers to the flow direction in ^1^D and ^2^D (*t*_fwd_) separation, and Valve B position refers to the flow direction in ^2^D separation (*t*_bwd_).

### Effect of Gradient Mobile Phase Ramp Rate

The two reverse-phase columns consisting of Water C18 and Agilent C18 were connected to the valve as the ^1^D and ^2^D-column, respectively. The suitable mobile phase was determined by separating crude wine W4 using five linear gradient ramp rates. The mobile phase was set at 10%(v/v) B for 5 min and gradually increased to 90%B at minute 20 (GMP1), 80 (GMP2), 120 (GMP3), 160 (GMP4), and 230 (GMP5). The details of each mobile phase system were described in Table S2. The valve switching times were set to *t*_fwd_ of 120 s and *t*_bwd_ of 90 s for FBF-VAST LC × LC, and a Valve A period of 30 s and Valve B period of 180 s for stop-flow LC × LC. The valve was activated 2 min after injection.

To determine the percentage increase in peak height resulting from the modulation mechanisms, we made a comparison between FBF-VAST LC × LC and stop-flow LC × LC. A mixture of five standard solutions, gallic acid, coumaric acid, benzoic acid, caffeine, and resveratrol at 50.00 mg/L, was investigated using GMP4 under the valve-switching conditions described above. The peak height of each standard was used to calculate the percentage increase observed with each system.

### Effect of Backward Flow Direction in the ^1^D-column

The FBF-VAST LC × LC mechanism, the backward flow direction (*t*_bwd_) was investigated in comparison to the normal flow direction (*t*_fwd_), following the flow arrow in Figure 1A. Separation was performed using *t*_fwd_ of 120 s and *t*_bwd_ of 90 s with GMP4 without ^2^D-column. The number of separable peaks in the HPLC chromatograms for both the normal and backward column directions was analyzed.

### Effect of Duration in Valve Position

The modulation period was fixed at 210 s while varying the valve switching times to *t*_fwd_ of 150 s and *t*_bwd_ of 60 s, *t*_fwd_ of 135 s and *t*_bwd_ of 75 s, *t*_fwd_ of 120 s and *t*_bwd_ of 90 s and, *t*_fwd_ of 114 s and *t*_bwd_ of 96 s, *t*_fwd_ of 111.25 s and *t*_bwd_ of 98.75 s, *t*_fwd_ of 107.5 s and *t*_*b*wd_ of 102.5 s. The optimal modulation period was determined based on the 2D plot separation space coverage and the number of separated peaks.

### Separated Peak Counting Procedure

The 1D modulated chromatograms were converted into 2D-control plots, and peak detection was performed using a Python-based algorithm employing image segmentation and contour analysis. Low-intensity peaks, wraparound features, and occasional false detections were subsequently corrected through manual verification.

### Gradient Mobile Phase Behavior in FBF-VAST in ^1^D-Column

Gradient mobile phase behavior in the ^1^D-column was monitored by a HPLC-UV–vis conventional method,^28^ with Milli-Q water as mobile phase A and acetone as mobile phase B without connecting the ^2^D-column. A blank (1.0 μL) was injected, and acetone in mobile phase B was detected at 265 nm, where it exhibits strong absorption.

### Investigation of Experimental Conditions

FBF-VAST was operated under different valve-switching times while maintaining a constant modulation period of 210 s. The experimental parameters included a gradient ramp from 10% to 90%B over varying durations and adjustments of the Valve A switching time. The number of separated peaks was counted using a Python-based algorithm combined with manual verification to assess the performance of each condition and select the most suitable condition.

### Method Validation

The repeatability of the FBF-VAST system was evaluated by analyzing the W4 sample in sextuplicate using GMP4 with valve switching times set at *t*_fwd_ of 114 s and *t*_bwd_ of 96 s. The peak area and retention time of the selected peaks were assessed.

The limits of detection (LOD) and quantification (LOQ) were determined using six standards (nicotinic acid, caftaric acid, vanillic acid, benzoic acid, *p*-coumaric acid, and caffeine) across concentrations ranging from 0.02 to 100.00 mg/L. The selected condition (GMP4), with valve switching times set to *t*_fwd_ = 114 s and *t*_bwd_ = 96 s), was applied. For each standard, the most intense modulated peak was used to calculate the corresponding LOD and LOQ values.

### Backflush Technique

Sample W8 was used in this study, and the separation process was performed under the same conditions as those mentioned in the LOD and LOQ analysis. The valve was activated at either 2 or 5 min after the introduction of the sample into the ^1^D-column to observe differences in separation performance. A backflush washing technique was applied by maintaining backward flow throughout the step after separation for 60, 40, or 20 min.

### Application

Benzoic acid, caffeine, caftaric acid, (+)-catechin, chlorogenic acid, (−)-epicatechin, nicotinic acid, *p*-coumaric acid, resveratrol, and vanillic acid, with concentrations ranging from 0.02 to 100.00 mg/L, were used as standards for qualitative and quantitative analysis of ten wine samples under conditions with *t*_fwd_ = 114 s and *t*_bwd_ = 96 s using GMP4.

Another RP column combination, consisting of a Waters C18 and a Shodex RSpak DE-613 (6.0 × 150 mm, 6.0 μm), was used as the ^1^D- and ^2^D-columns to compare the separation performance with the Waters C18 and Agilent C18 systems using the DCM wine extract (W6) and PW6. The separation was performed under conditions of *t*_fwd_ = 120 and *t*_bwd_ = 90 s using GMP4 and GMP6, and *t*_fwd_ = 110 and *t*_bwd_ = 100 s using GMP6.

## Theoretical Background

The migration of each analyte along the column can be modeled by using an advection-dispersion equation modified to incorporate retention effects:

1 $$\frac{\partial C\left(x,t\right)}{\partial t}+\frac{\partial }{\partial x}\left(v_{\mathrm{eff}}\left(x,t\right)C\left(x,t\right)\right)=D\frac{\partial^{2}C\left(x,t\right)}{\partial x^{2}}$$

where *C*(*x*,*t*) is the concentration of analyte at position *x* and time *t*, and *D* is the apparent axial diffusion coefficient. The effective migration velocity, *v*_eff_(*x*,*t*), accounts for analyte retention through the retention factor *k*(*x*,*t*), given by

2 $$v_{\mathrm{eff}}\left(x,t\right)=\frac{v_{\mathrm{flow}}}{1+k\left(x,t\right)}$$

where *v*_flow_ is the bulk mobile phase velocity. For gradient-dependent retention, a linear-log model is used for *k*(*x*,*t*) calculation as

3 $$\log_{10}k\left(x,t\right)=-a\cdot B\left(x,t\right)+b\Rightarrow k\left(x,t\right)=10^{-a\cdot B(x,t)+b}$$

where *B*(*x*,*t*) represents the local mobile phase composition (%B). The parameters *a* and *b* are empirically determined retention coefficients specific to each analyte. Since *k*(*x*,*t*) depends on *B*(*x*,*t*), accurately modeling the spatiotemporal evolution of the mobile phase composition along the column is essential for capturing analyte transport dynamics, as governed by

4 $$\frac{\partial B\left(x,t\right)}{\partial t}+\upsilon_{\mathrm{flow}}\frac{\partial B\left(x,t\right)}{\partial x}=D_{B}\frac{\partial^{2}B\left(x,t\right)}{\partial x^{2}}$$

where *B*(*x*,*t*) is the concentration of the mobile phase at position *x* and time *t*, *D*_B_ is the effective diffusivity for the mobile phase front. A linear gradient ramp is imposed at the column inlet according to

5 $$B_{\mathrm{inlet}}\left(t\right)=\{\begin{array}{ll} B_{\mathrm{init}}, & {t<t}_{\mathrm{hold}}\\ B_{\mathrm{init}}+\left(B_{\mathrm{final}}-B_{\mathrm{init}}\right)\cdot \frac{t-t_{\mathrm{hold}}}{t_{\mathrm{ramp}}}, & t_{\mathrm{hold}}\leq {t<t}_{\mathrm{ramp}\text{\_}\mathrm{end}}\\ B_{\mathrm{final}}, & t\geq t_{\mathrm{ramp}\text{\_}\mathrm{end}} \end{array}$$

In the FBF-VAST system, periodic flow reversal modulation is implemented by alternating the flow direction over time according to

6 $$\mathrm{direction}\left(t\right)=\{\begin{array}{ll} \mathrm{forward}, & \mathrm{if}\;t<t_{\mathrm{init}}\;\mathrm{or}\;(t-t_{init})\%T_{\mathrm{cycle}}<T_{\mathrm{fwd}}\\ \mathrm{backward}, & \mathrm{otherwise} \end{array}$$

This was applied using a total modulation cycle of *T*_cycle_ = *T*_fwd_ + *T*_bwd_.

The boundary and initial conditions were defined as follows: the initial mobile phase composition was uniform along the column, expressed as

7 $$B\left(x,0\right)=B_{init}$$

At the inlet, the boundary condition was defined as

8 $$B\left(0,t\right)=B_{inlet}\left(t\right)$$

At the outlet, a Neumann-type or one-sided extrapolation condition was applied. The initial analyte distribution was represented by a Gaussian peak centered at *x*_0_ = 5 mm at *t* = 0:

9 $$C\left(x,0\right)=\exp\left(-\frac{(x-x_{0}{)}^{2}}{{2\sigma }^{2}}\right)$$

For analyte transport, nonreflective (open) boundary conditions were assumed, allowing loss at both ends of the column. The simulation was conducted under the following assumptions: 1) ^1^D axial transport is sufficient, so that radial diffusion can be neglected, 2) solute-stationary interaction is modeled only via *k* without explicit adsorption, 3) no chemical reactions or degradation during transport, 4) mobile phase is Newtonian, incompressible, and well-defined by %B, and 5) flow reversal is instantaneous and does not disrupt the system’s pressure or composition continuity.

To emulate realistic detector behavior and extract analyte and mobile phase signals, the net component loss at the outlet is computed at each time step: when the flow is forward, the detector is positioned at *x* = *L* = 150 mm (at the column end adjacent to the detector) according to

10 $${\mathrm{signal}}_{\mathrm{fwd}}\left(t\right)=\max\left[0,C\left(t-1,x-1\right)-C\left(t,x\right)\right]$$

and when the flow is reversed, the detector is located at *x* = 0 mm (at the column end adjacent to the injector) according to

11 $${\mathrm{signal}}_{\mathrm{bwd}}\left(t\right)=\max\left[0,C\left(t-1,x+1\right)-C\left(t,x\right)\right]$$

To this end, the “column outlet” is defined as the end where the mobile phase exits toward the detector (either at *x* = 150 mm or *x* = 0 mm, depending on valve operation), while the “column inlet” refers to the end where the mobile phase enters from the injector (either at *x* = 0 mm or *x* = 150 mm, also depending on valve operation at that time). Detection of the mobile phase followed the same principles as described in eqs 10–11, with parameter *C* replaced by parameter *B* to represent the mobile phase composition.

## Results and Discussion

### Overview of FBF-VAST as a New Modulation Approach

The newly developed forward–backward-flushing valve-assisted selectivity tuning (FBF-VAST) system represents a unique approach to modulation in LC × LC. Although it does not utilize conventional dual-loop, thermal, or trapping column designs, FBF-VAST achieves modulation through the dynamic switching of flow direction within the ^1^D-column using a single 4-port, dual-position valve (Figure 1A). This mechanism allows for timed fractionation of the ^1^D effluent and controlled forward reinjection onto the ^2^D-column, thereby fulfilling the key functional criteria of modulation: analyte slicing, transfer, and refocusing. All experimental conditions applied to the figures, as well as the rationale behind the experimental design, are summarized in the Supporting Information.

FBF-VAST presents several notable advantages. Its operational simplicity eliminates the need for multiple pumps, trapping devices, or cryogenic modules, while still achieving pseudofocusing effects through precise flow manipulation. The system also enables selectivity tuning by adjusting the valve operations. This conceptual study utilized a reversed-phase × reversed-phase (RP × RP) configuration to highlight the impact of selectivity tuning. Notably, the system can be expanded with solvent exchange modules to facilitate separations employing different ^1^D and ^2^D separation modes. However, this requires additional equipment, such as solvent exchange modules or auxiliary pumps. Furthermore, the back-flush process improves peak transfer efficiency and can enhance analyte enrichment under gradient elution conditions with increasing mobile phase strength. Under gradient conditions, increasing the mobile-phase strength reduces analyte retention and promotes migration along the column. In the FBF-VAST configuration, the backward-flow step can temporarily retain the analyte until a stronger mobile phase (higher %B) is reached. The subsequent forward–backward cycling effectively sweeps and compresses the analyte into a narrower band prior to detection, which resulted in enhanced signal intensity and signal-to-noise ratio, improving LOD and LOQ. This enrichment effect is illustrated in Figure S2, where FBF-VAST produces sharper and more intense peaks compared with conventional 1D-LC. It is also capable of backflushing the ^1^D-column, allowing for automated online column cleaning following analysis. Overall, FBF-VAST offers a compelling balance between system simplicity and modulation performance, positioning it as a novel alternative to both passive and active modulation strategies in LC × LC.

### Principle and Modulation Mechanism

One approach to modulation involves longer forward–shorter backward valve switching, where segments of the effluent from the ^1^D-column are redirected by alternating the flow direction. Under gradient elution conditions, however, this system behaves nontrivially. A mobile phase gradient propagates from the injector through the first column, but valve switching introduces complex mobile phase dynamics. In this study, we initially focused on the simulation and experimental observation of the axial %B profile changes along the ^1^D-column during such modulation, aiming to clarify how mobile phase composition dynamically redistributes, particularly during asymmetric valve cycles.

### Simulation of Mobile Phase Gradient Dynamics in FBF-VAST

To investigate the spatiotemporal behavior of the mobile phase gradient in the ^1^D-column under alternating flow conditions, a numerical simulation was conducted based on eq 4. With the given inlet profile of %B in eq 5, the simulation aimed to elucidate the temporal evolution of %B along the column axis and to correlate the simulated profiles with the experimentally observed signals. While this inherently models the % B entering the second-dimension column, the simulation of the ^2^D separation outcomes is beyond the scope of this study and was therefore not undertaken.

A binary mobile phase system composed of water (A) and acetone (B) was used, in which the initial mobile phase consisted of 10% acetone for the first 5 min, followed by a linear gradient increase to 90% acetone at 160 min. The column used was 150 mm in length with an internal diameter of 3.9 mm and a particle size of 4 μm. A constant flow rate of 0.5 mL/min was maintained throughout the simulation.

Valve programming was configured as follows: an initial forward flow (Valve A) was applied for 120 s, starting at *t* = 0. From *t* = 120 s onward, the system alternated between forward flow (Valve A, 120 s) and backward flow (Valve B, 90 s) in a repeating cycle (eq 6). This simulates a modulation sequence, where mobile phase gradients are shuffled back and forth within the primary column. *D* for acetone in water at room temperature was approximated as 1.2 × 10^–5^ cm^2^/s.^29^ The velocity was derived from the volumetric flow rate and column cross-sectional area. The numerical solution of eq 4 was implemented by using a finite-difference method with first-order upwind discretization for the convection term and second-order central differences for the diffusion term. The axial domain was divided into 10001 grid points, and time was discretized with a step of 0.1 s. Boundary conditions were time-dependent, adjusted at each valve-switching event, and the mobile phase input profiles were updated accordingly to reflect either forward or backward flow (eqs 6–8). During Valve A operation (forward flow), the inlet at 0 mm receives the current %B from the gradient program, while the outlet at 150 mm is treated as convective. Conversely, during Valve B operation (backward flow), the gradient is applied at 150 mm (now the inlet), and the outlet at 0 mm becomes convective. In both cases, the outlets are assumed to be directly connected to the detector without dead volume in the simulation.

Simulation results were visualized as a heatmap of %B versus position and time, using a colormap to distinguish between low (dark blue) and high (yellow) acetone content (Figure 1B). An animation showing the dynamic evolution of the mobile phase profile is provided in Supporting Information. To approximate the chromatographic profile, a simulated detector signal was extracted over time (Figure 1C). For validation, this simulation was compared with experimentally recorded profile fluctuations (Figure 1D) using acetone as a UV-active tracer in the mobile phase because acetone has similar physiochemical properties to acetonitrile and less toxicity than methanol. The main strength is the UV cutoff at 265 nm of acetone, which is higher than that of water, which is also used as a mobile phase in this work. This enables mobile phase behavior tracking during valve operation at this specific wavelength.^28,30^ The system was configured as injector → ^1^D-column → detector, excluding the ^2^D-column to isolate the first-column solvent behavior. Real-time acetone absorbance was monitored at the detector, while modulation was controlled by periodic valve switching.

Both simulation and experimental results display similar trends and %B profile shapes. However, differences in signal amplitude may arise due to system factors not included in the simulation—such as a transient backpressure increase during valve switching or tubing void volume effects between the column outlet and the detector. Detailed analysis of the simulation revealed that, during forward flow (Valve A), the %B gradient is progressively established along the column. During backward flow (Valve B), the gradient profile becomes spatially spread and partially mixed due to the inversion of the flow direction. Because of this alternating forward–backward modulation with the increasing %B program, the detector signal exhibits a series of stepped gradients. Within each step, a slight negative slope is observed (indicated by red arrows in Figure 1C), attributed to the backward flow flushing lower-%B segments back toward the detector.

In the studied conditions, the forward flow period (*t*_fwd_) was longer than the backward period (*t*_bwd_). As a result, the forward-moving %B step is fully flushed through the 150 mm outlet and detected. However, the backward-moving step is only partially displaced, leaving behind what can be thought of as “*half a pond*” of %B within the column near the 0 mm outlet (see the animation in the Supporting Information). When the flow direction switches back to Valve A, a new higher %B segment is introduced at the 0 mm inlet and combines with the remaining (*half a pond*) portion of the previous segment—forming a “*full pond*” that is then flushed toward the detector.

This alternating pattern of “*half a pond* moving backward” and “a *full pond* moving forward” repeats throughout the modulation, producing the characteristic signal dips observed in each modulation cycle in Figure 1C. These dips occur at the end of each Valve A period, followed by sharp increases in the signal upon switching to Valve B, when a higher-%B segment at the 0 mm outlet is suddenly flushed to the detector. Our revised interpretation clarifies that the first signal dip is observed not during the first Valve A cycle but during the second and subsequent cycles—reflecting the point at which a complete %B segment is first introduced into the detection path.

### Simulation of Analyte Modulation in FBF-VAST

The simulated mobile phase space–time profiles were then used to calculate the local retention factor *k*(*x*, *t*) at each position and time point based on eqs 2 and 3. When combined with eq 1, the same numerical simulation framework was extended to model the analyte modulation profiles. With the given initial analyte distribution (eq 9), simulations were performed for gallic acid, caffeine, resveratrol, *p*-coumaric acid, and benzoic acid using their respective *a* and *b* parameters in eq 3, which were experimentally determined via 1D-LC analysis on the primary column only. The extracted parameters were 0.005 and 0.45 for gallic acid, 0.019 and 1.32 for caffeine, 0.024 and 1.60 for resveratrol, 0.025 and 1.30 for *p*-coumaric acid, and 0.030 and 1.30 for benzoic acid. The FBF-VAST process generates analyte profiles that effectively mimic postcolumn modulation following elution from the ^1^D, as illustrated for five representative analytes in Figure 2. For clarity, a step-by-step simulation of the modulation process for resveratrol is provided in Figure S3 with the corresponding analyte movement further illustrated in Video S1. This figure captures the temporal evolution of a single analyte during successive forward–backward flushing cycles, showing both spatial peak profiles along the column and the corresponding detector signals at discrete time points (Figure S3A–H). It illustrates how the analyte is periodically displaced, retained, and released, ultimately giving rise to discrete modulated signals. The migration behavior of all analytes is further visualized as an overlay of space–time–intensity maps in Figure S4, providing a comprehensive view of their separation trajectories. Animations depicting the temporal evolution of analyte profiles are also included in Supporting Information, Video S2. In addition, the use of identical stationary phases under gradient conditions does not preclude orthogonality in multidimensional separations. A similar phenomenon has been reported in temperature-programmed GC × GC using a single stationary phase, where effective orthogonality arises from differences in thermal conditions experienced by analytes in each dimension.^31^ Although the same phase is used, analytes encounter distinct temperature windows in the first and second dimensions, leading to different retention behaviors due to the nonlinear and compound-specific dependence of retention on temperature. An analogous principle applies in LC × LC. Despite employing the same stationary phase and mobile-phase system, analytes experience different solvent-strength environments (%B) in each dimension due to the time offset introduced by modulation. Because retention in LC depends nonlinearly on solvent composition, this results in effectively different selectivity windows between dimensions, thereby enabling orthogonality. Furthermore, the modulation process enhances separation by slicing broader 1D peaks into narrower subpeaks prior to second-dimension analysis. This reduces the local analyte concentration and improves the resolution of closely eluting compounds. Together, these effects demonstrate that effective two-dimensional separation can be achieved even with identical stationary phase and gradient conditions.

**Figure 2:**
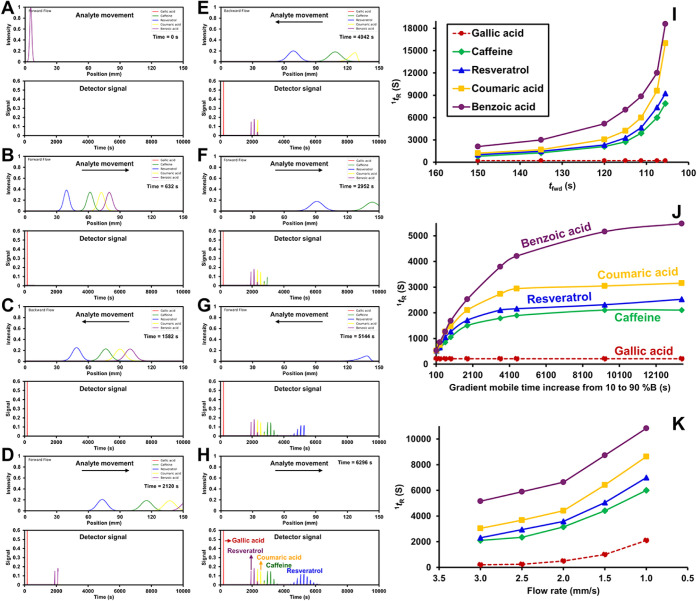
Simulated analyte peak profiles (above) and corresponding detector signals (below) under FBF-VAST modulation, using only the ^1^D Waters C18 column. Results are shown for gallic acid, caffeine, resveratrol, coumaric acid, and benzoic acid at different times following injection (A-H, respectively). Demonstration of selectivity tuning is also simulated by variation of ^1^ *t*_R_ for five analytes (gallic acid, caffeine, resveratrol, *p*-coumaric acid, and benzoic acid) under different experimental parameters in FBF-VAST: decreasing *t*_fwd_ (I), increasing gradient time from 10% to 90% B (J), and decreasing flow rate during the first-dimension separation (K).

Under FBF-VAST operation, both the gradient and the analyte peaks were visibly modulated and redistributed within the primary column across multiple modulation cycles. This confirms that the mechanism allows dynamic modulation of both the solvent composition and analyte location in a time-dependent, controllable manner. To this end, the modulation period (*P*_M_) could be defined by *t*_bwd_ + *t*_fwd_, with the net injection window (Δ*t*_net_ = *t*_fwd_ – *t*_bwd_) transferring analyte pulses onto the ^2^D-column.

The FBF-VAST modulation mechanism allows analytes to be temporarily retained on the ^1^D-column before being transferred to the second dimension. Consequently, the %B profile experienced by analytes in both dimensions depends not only on their intrinsic *t*_R_ but also on the timing of Valve A (*t*_fwd_) and Valve B (*t*_bwd_). By adjusting the durations of these valve periods, users can alter when and how analytes are exposed to higher or lower %B conditions, effectively tuning the selectivity.

This effect is demonstrated in the simulated ^1^ *t*_R_ vs *t*_fwd_ plot (Figure 2I), where decreasing *t*_fwd_ generally results in longer ^1^ *t*_R_ with the late-eluting peaks showing more compressed apparent ^1^ *t*_R_ (shifting closer together). Importantly, this selectivity behavior contrasts with that produced by changing the gradient slope (Figure 2J). A shallower gradient also causes ^1^ *t*_R_ to increase overall until leveling off with the late-eluting peaks exhibit more widely spaced apparent ^1^ *t*_R_ (shifting further apart).

This contrasting behavior arises from the FBF-VAST mechanism, in which analytes are retained on the column under progressively stronger (%B-rich) conditions during the forward-flow modulation cycle. As a result, later-eluting compounds are exposed to %B values higher than those they would experience under a conventional gradient. It is also worth noting that selectivity tuned by *t*_fwd_ differs from that obtained through flow-rate variation, which induces only minor changes in selectivity (Figure 2K). Furthermore, the tunability of analyte ^1^ *t*_R_ can lead to different %B exposure profiles in the ^2^D separation since the same mobile-phase gradient program was applied in both dimensions.

### Experimental Selectivity Tuning in FBF-VAST

To experimentally demonstrate this selectivity-tuning capability, a FBF-VAST LC × LC system was configured using two conventional reversed-phase C18 columns for the separation of a wine sample (W4). The first dimension used a Waters AccQ-Tag C18 column (34 resolved peaks in 1D), while the second dimension employed an Agilent ZORBAX Eclipse Plus C18 column (29 resolved peaks under stand-alone 1D conditions). This setup allowed for direct assessment of how the FBF-VAST valve timings influence peak distribution and selectivity in two-dimensional space. It should also be noted that backward flow directions have generally not been recommended due to concerns that they may cause column damage, lead to an unsymmetrical particle distribution within the column, and reduce the theoretical plate count, among other potential issues. However, backward flow direction is also a recommended technique for column cleanup, known as “Backflushing.” This method reverses the flow direction by switching the valve position to elute untargeted analytes after collecting the target analytes.^32,33^ It should be noted that although the backflush flow is performed under controlled conditions, the continuous alternation between forward and backflush cycles may affect the organization of the stationary phase particles, potentially impacting the column’s lifetime.

Given the FBF-VAST system has forward and backward flushing in the^1^D-column when valve positions change,^34^ the column was connected to the valve for a 2D system without a ^2^D-column connected. Forward and backward flows in the ^1^D-column can be initiated from either Valve A or Valve B. The system was operated under either exclusively forward or backward flow, controlled by the valve positions, to assess the impact of reverse flow compared to the normal forward-flow direction. The results indicated consistent chromatograms (Figure S5), with the number of separated peaks being 29 for the normal flow direction and 28 for the backward flow direction. The results indicate that the column flow direction with different column entries has a negligible effect on column performance. This may be due to the homogeneous packing process and symmetrical column design at both frit ends, preventing heterogeneity and particle loss under high pressure.^35^

### Effect of Gradient Mobile Phase Ramp Rate

The effect of different gradient ramp rates was investigated using valve durations set at a *t*_fwd_ of 120 s and *t*_bwd_ of 90 s. This corresponds to a *P*_M_ of 210 s and the ^1^D to ^2^D pulse transfer duration of 30 s. The results are shown in Figure 3A–E. The relationship between the number of separated peaks in the 2D control plot results (Figure 3F) was used to determine the appropriate gradient mobile phase. In FBF-VAST, under GMP1 and GMP2 conditions, the mobile phase was too fast to achieve adequate separation, as shown in Figure 3A–B with most of the compounds eluting at the lower ^1^ *t*_R_. The fast gradients also reduced retention, resulting in low-quality ^2^D separation. The separation performance improved when the mobile phase increased more gradually (GMP3–5), as shown in Figure 3C–E, resulting in a higher number of separable peaks and improved resolution. GMP4 (Figure 3D) and GMP5 (Figure 3E) demonstrate good separation and distribution in 2D space, with the number of separated peaks being 57 and 64, respectively. However, GMP5 required a longer separation time compared to GMP4 and resulted in lower intensities of peaks. Therefore, GMP4 was sufficiently effective for separating an approximate number of compounds and was selected for further studies.

**Figure 3:**
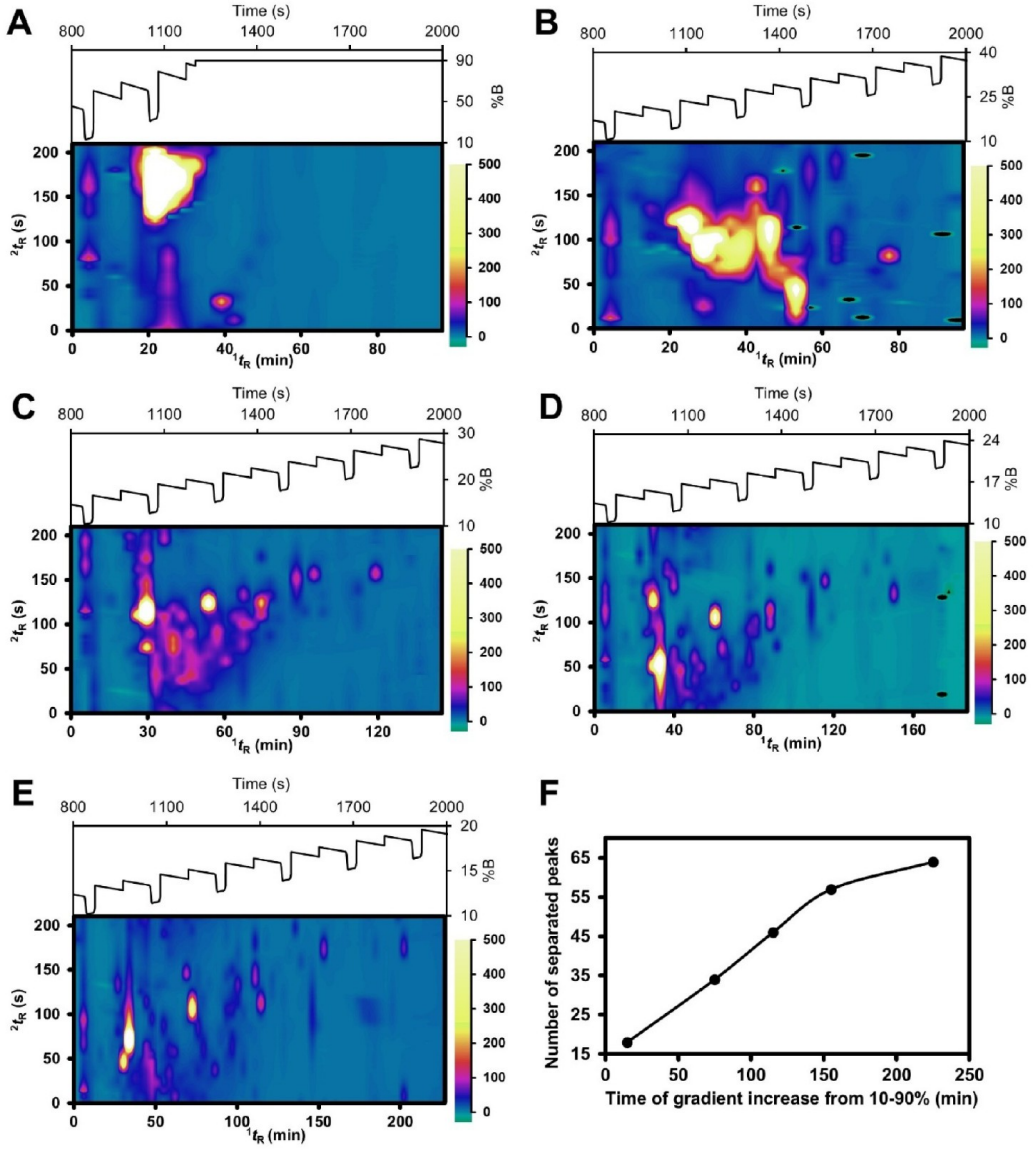
Control plots showing the effect of gradient mobile phase ramp rate in FBF-VAST LC × LC at GMP1–5 (A–E, fastest to slowest gradient, respectively) using *t*_fwd_ of 120 s and *t*_bwd_ of 90 s. Scales are identical in all 2D-control plots. The corresponding plot of the number of separated peaks versus the gradient conditions is shown in F.

It should be noted that the separation performance of the developed FBF-VAST (Figure S6A) method was compared with that of the stop-flow modulation under GMP3 (Figure S6B). The stop-flow approach was performed using the same 4-port valve albeit with different connections as shown in Figure S1 and the valve durations were set at Valve A period of 180 s and Valve B period of 30 s in order to result in the same modulation period (210 s) and the ^1^D to ^2^D pulse transfer duration (30 s). The number of separated peaks obtained with the FBF-VAST system was slightly higher (46 peaks) than that achieved under stop-flow conditions (44 peaks). A key advantage of FBF-VAST is the reduced pressure fluctuation, as demonstrated in Figure S7, together with an enhanced selectivity-tuning capability. In addition, FBF-VAST provides more effective utilization of the two-dimensional separation space, resulting in improved peak distribution across the 2D contour plot compared to the stop-flow approach (Figure S6). In contrast, the stop-flow method exhibited higher signal intensity (by approximately 1.2–1.7×), indicating a degree of sensitivity loss in FBF-VAST. This effect can be attributed to the unconventional %B profile introduced during the modulation process (Figure 1D), which influences analyte focusing in the second dimension. Nevertheless, this limitation can be mitigated by reducing the *P*_M_ to improve band compression or by incorporating a secondary mobile-phase stream and/or a trapping interface to enable solvent exchange toward a more conventional %B profile in the second dimension. Such modifications conceptually align with active modulation strategies, while retaining the simplicity and flexibility of the FBF-VAST design.

### Effect of Duration in Valve Position (Separation Result Tuning by Changing*t*_fwd_)

Figure 4 shows the separation tuning results obtained from varying the valve position durations in the FBF-VAST LC × LC system under the same gradient condition, GMP4. The modulation period was fixed at 210 s. Different durations of forward and backward valve positions (*t*_fwd_ and *t*_bwd_) significantly affected the separation efficiency. In this section, we refer only to *t*_fwd_ since *t*_bwd_ could be expressed as 210-*t*_fwd_. In general, the longer *t*_fwd_ could earlier transfer pulses onto the ^2^D-column leading to stronger ^2^D retention due to the separation occurring at lower %B. An extreme case could be illustrated in Figure 4A where only forward flow was applied (without modulation), resulting in the strong wraparound result. On the other hand, too short *t*_fwd_ held compounds onto the ^1^D-column longer during the modulation leading to weaker ^2^D retention due to the separation occurring at higher %B. The extreme case could be illustrated in Figure 4G with the overall lower peak ^2^ *t*_R_ and strong coelution of compounds. The optimal condition was observed when using *t*_fwd_ of 120 s, which provides a balance between the strong ^2^D retention and the small pulse transfers (Figure 4D).

**Figure 4:**
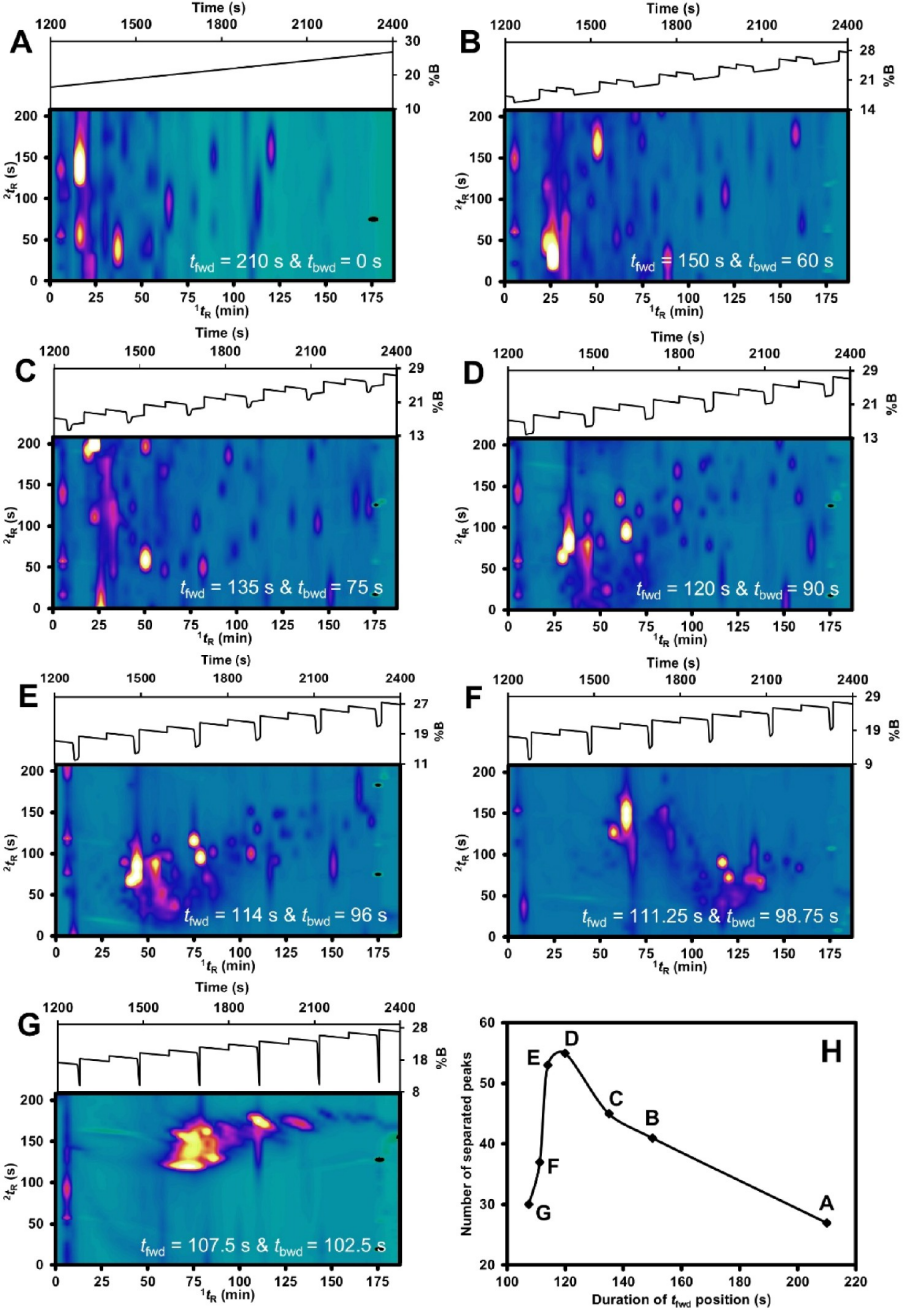
2D-plot of *t*_fwd_ for the whole duration (A), six different *t*_fwd_ durations (B-G) with the modulation period fixed at 210 s using GMP4, and the correlation plot between the number of separated peaks and the duration of *t*_fwd_ position (H).

Application of *t*_fwd_ = 120 s and *t*_bwd_ = 90 s under the GMP4 condition produced the largest number of separable peaks in the contour plot (Figure 4D), demonstrating effective separation and comprehensive coverage of the two-dimensional space. However, this setting also exhibited a substantial degree of wraparound. To mitigate this effect, *t*_fwd_ was slightly reduced to allow compounds to elute on the second column under stronger mobile-phase conditions (later in the gradient). Consequently, the condition with *t*_fwd_ = 114 s and *t*_bwd_ = 96 s (Figure 4E), which yielded the second-highest number of separable peaks without a noticeable wraparound, was selected for subsequent analyses.

### Method Validation

The FBF-VAST LC × LC results were validated by comparison with the 1D-LC technique. Key analytical parameters including LOD, LOQ, linearity range, and repeatability were evaluated under the selected GMP4 condition with *t*_fwd_ = 114 s and *t*_bwd_ of 96 s.

Six standards, nicotinic acid, caftaric acid, vanillic acid, caffeine, *p*-coumaric acid, and benzoic acid, were used for the evaluation, with the results summarized in Table 1. The FBF-VAST LC × LC method exhibited lower LODs (0.02–0.05 mg/L) and LOQs (0.06–0.18 mg/L) compared to the 1D-LC method (LOD: 0.05–0.13 mg/L; LOQ: 0.15–0.39 mg/L). The only exception was nicotinic acid, for which both methods produced comparable LOD and LOQ values. Both techniques demonstrated good linearity, with the FBF-VAST LC × LC system achieving R^2^ > 0.98 and the 1D-LC method achieving R^2^ > 0.98.

**Table 1:** Validation Results of the FBF-VAST LC × LC Method Using GMP4, *t*_fwd_ = 114 s, and *t*_bwd_ of 96 s Compared with 1D-LC Using GMP4 and an AccQ-Tag Column^a^

	FBF-VAST LC × LC				1D-LC			
Standard compounds	Equation	R^2^	LOD	LOQ	Equation	R^2^	LOD	LOQ
Nicotinic acid	y=69.6x+37.9	0.998	0.05	0.15	y=68.8x+7.9	0.982	0.05	0.15
Caftaric acid	y=161.9x+150.8	0.997	0.05	0.15	y=161.9x+150.8	0.997	0.10	0.30
Vanillic acid	y=155.7x+85.3	0.995	0.03	0.18	y=138.5x−31.7	0.994	0.13	0.39
Caffeine	y=168.2x−159.7	0.997	0.02	0.06	y=155.4x+8.1	0.993	0.10	0.30
*p*-Coumaric acid	y=275.7x−381.2	0.989	0.05	0.20	y=265.0x−100.6	0.982	0.12	0.34
Benzoic acid	y=76.7x−195.1	0.989	0.04	0.13	y=79.0x+11.1	0.980	0.05	0.15

a The instrument LOD and LOQ are expressed in mg/L.

Sextuplicate results of five peaks from the FBF-VAST chromatograms were obtained to evaluate the precision of the developed method. Peak identities were confirmed using spectral scanning mode on the DAD detector, with an example provided in Figure S8 and Table S3. The FBF-VAST analysis approach demonstrated acceptable repeatability, with precision of peak area within 5.28–11.77 %RSD.

### Backflush for Washing Techniques

In this study, a backflushing strategy was applied to reduce the overall analysis time by eliminating matrix components following the designated separation period. Backflush washing was initiated by switching the valve to reverse the flow direction after the completion of the separation period. Various valve activation times were examined to optimize the backflush operation and enhance the overall separation performance. Valve activation at 5 min resulted in enhanced resolution, as shown in the W8 2D-plot in Figure 5C, compared with activation at 2 min (Figure 5A). Based on this observation, we assumed that once the target analytes are fully separated within 60, 40, or 20 min, backflush washing can subsequently be applied. As illustrated in Figure 5B, D and E, the back-flush step produces a large peak following the switch to reverse flow, indicating that compounds remaining at the column inlet are efficiently removed rather than retained within the column. This approach produced a noticeably cleaner baseline compared to analyses without backflush washing. Under the selected mobile-phase conditions, the backflush step reduced the total analysis time by more than 40 min (from 190 to 150 min) while maintaining a clean and reliable chromatogram.

**Figure 5:**
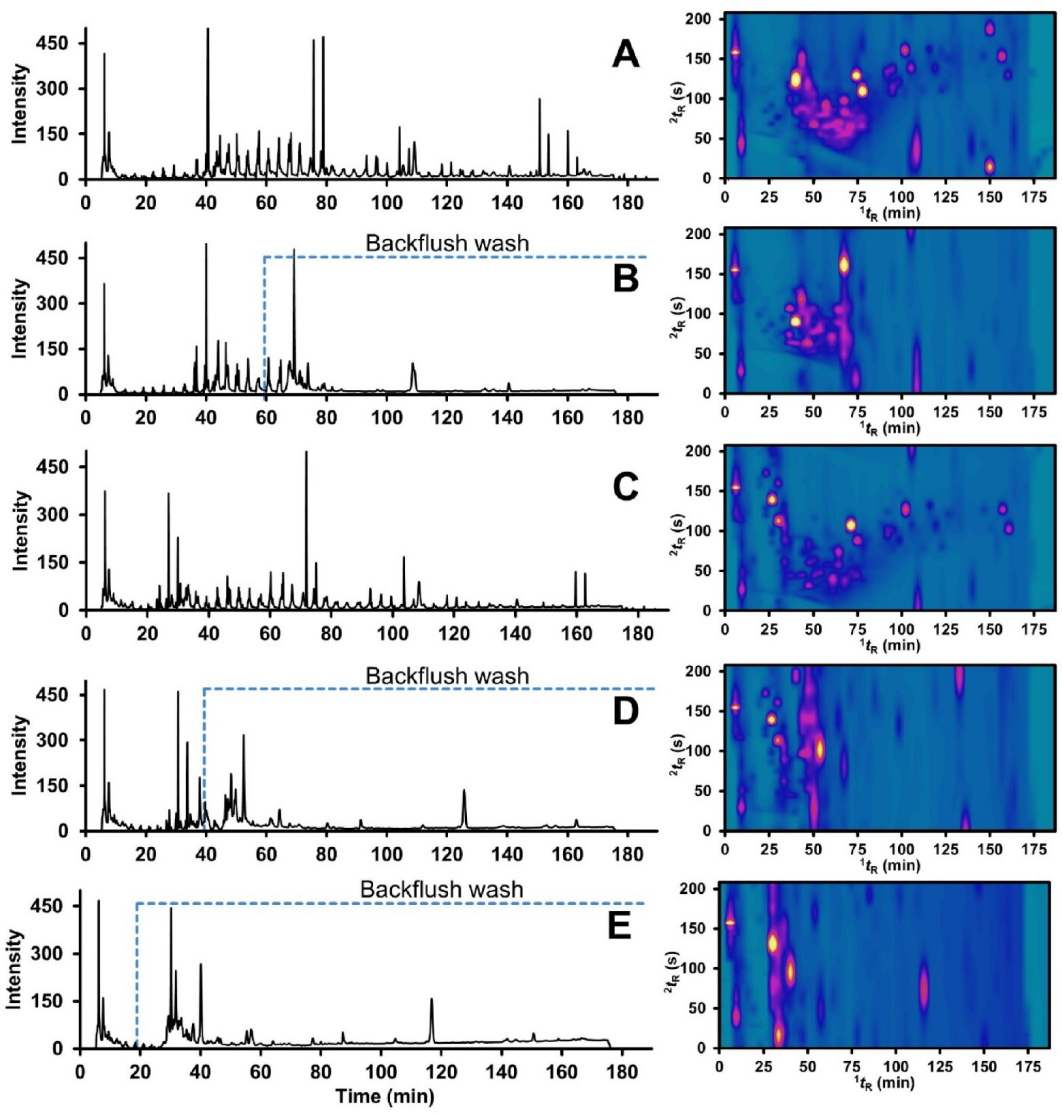
1D-modulated chromatograms and 2D-control plots of backflushing studies with the valve starting time at 2 min: nonbackflush washing (A), backflush washing after 60 min (B), and the valve starting time at 5 min: nonbackflush washing (C), backflush washing after 40 min (D), and 20 min (E).

### Application

The selected FBF-VAST LC × LC condition (GMP4 with *t*_fwd_ of 114 s and *t*_bwd_ of 96 s) was applied to the qualitative and quantitative analysis of 10 mixed standards in 10 DCM wine extract samples. Standards were divided into three groups consisting of phenolic acids (benzoic acid, caftaric acid, chlorogenic acid, *p*-coumaric acid, and vanillic acid), flavonoids ((+)-catechin and (−)-epicatechin) and others including caffeine, nicotinic acid, and resveratrol.

The peaks in the LC × LC results were identified by comparing retention times at 210 nm and UV spectra (under the scan mode of the DAD) to those of the standards. For quantitative analysis, calibration curves were constructed for the target compounds within the concentration range of 0.02–100 mg/L, as shown in Table S4, along with the corresponding linear equation for each standard.

Three types of wine, 7 red wines (Shiraz), 1 white wine (Riesling), and 2 fruit wines (Lychee or Mangosteen), were analyzed using the optimized method. The number of separated target analytes appeared to be strongly influenced by the type of fruit, with red wines (Shiraz) generally exhibiting the highest number of compounds, followed by fruit wines made from Lychee and Mangosteen and white wine (Riesling). As shown in Table S4, *p*-coumaric acid was the most widely detected compound, present in all samples except fruit wine W10. Among red wines, *p*-coumaric acid exhibited the highest levels (0.017–0.066 mg/g), followed by white wine W8 (0.015 mg/g) and fruit wine W9 (0.008 mg/g). Caftaric acid was detected in the Thai red wines (W3–W6), with concentrations ranging from 0.002–0.008 mg/g, but it was absent in W7 and in the red wines from Australia (W1) and France (W2). Higher levels were observed in white wine W8 (0.022 mg/g), and moderate levels were observed in fruit wine W9 (0.004 mg/g). Chlorogenic acid was predominantly detected in red wines, with concentrations of 0.013 mg/g (W4) and 0.016 mg/g (W5), whereas vanillic acid was present at lower levels, ranging from 0.001–0.003 mg/g across samples W3–W4 and W6–W7. Benzoic acid was found exclusively in fruit wine W10 at a concentration of 0.016 mg/g.

The flavonoids (+)-catechin and (−)-epicatechin were predominantly detected in red wines, with (+)-catechin present at relatively high levels in W2–W4 and W6 (0.002–0.044 mg/g), and in the fruit wine W9 (0.011 mg/g). It was not detected in white wine or fruit wine W10. In contrast, (−)-epicatechin was observed in white wine and both fruit wines at 0.002 and 0.004–0.005 mg/g, respectively, and in red wines W4–W7 at 0.002–0.006 mg/g.

Among other compounds, nicotinic acid and resveratrol were predominantly detected in red wines and were not detected in white wine. Nicotinic acid was present in all red wines (0.004–0.013 mg/g) and fruit wines (0.008–0.011 mg/g). Resveratrol exhibited a distribution similar to that of nicotinic acid, except that it was not detected in fruit wine W10. Its concentration ranged from 0.009 to 0.039 mg/g in red wines and was 0.010 mg/g in fruit wine W9. Caffeine was not detected in the analyzed samples.

Apart from the same stationary-phase combination above (^1^D Waters C18 × ^2^D Agilent C18), an FBF-VAST LC × LC system using two different RP columns (^1^D Waters C18 × ^2^D RSpak DE-613) was also employed to compare separation performance for the separation of a DCM wine extract (W6). Figure 6A shows the contour plots obtained using the ^1^D Waters C18 × ^2^D Agilent C18 system (GMP4 with *t*_fwd_ = 120 s and *t*_bwd_ = 90 s) for the red wine extract. The separation exhibits a good peak distribution and efficient utilization of the 2D separation space. Even with the similar stationary-phase chemistry and comparable column dimensions, the developed FBF-VAST approach provided sufficient selectivity tuning capability, allowing separation of most analytes within the modulation window without significant wraparound. In comparison, the ^1^D Waters C18 × ^2^D RSpak DE-613 system (Figure 6B) also demonstrates good separation coverage but shows noticeable wraparound behavior. This effect can be attributed to more significantly different selectivities in ^1^D and ^2^D separations by using different stationary phases combined with the FBF-VAST mechanism.

**Figure 6:**
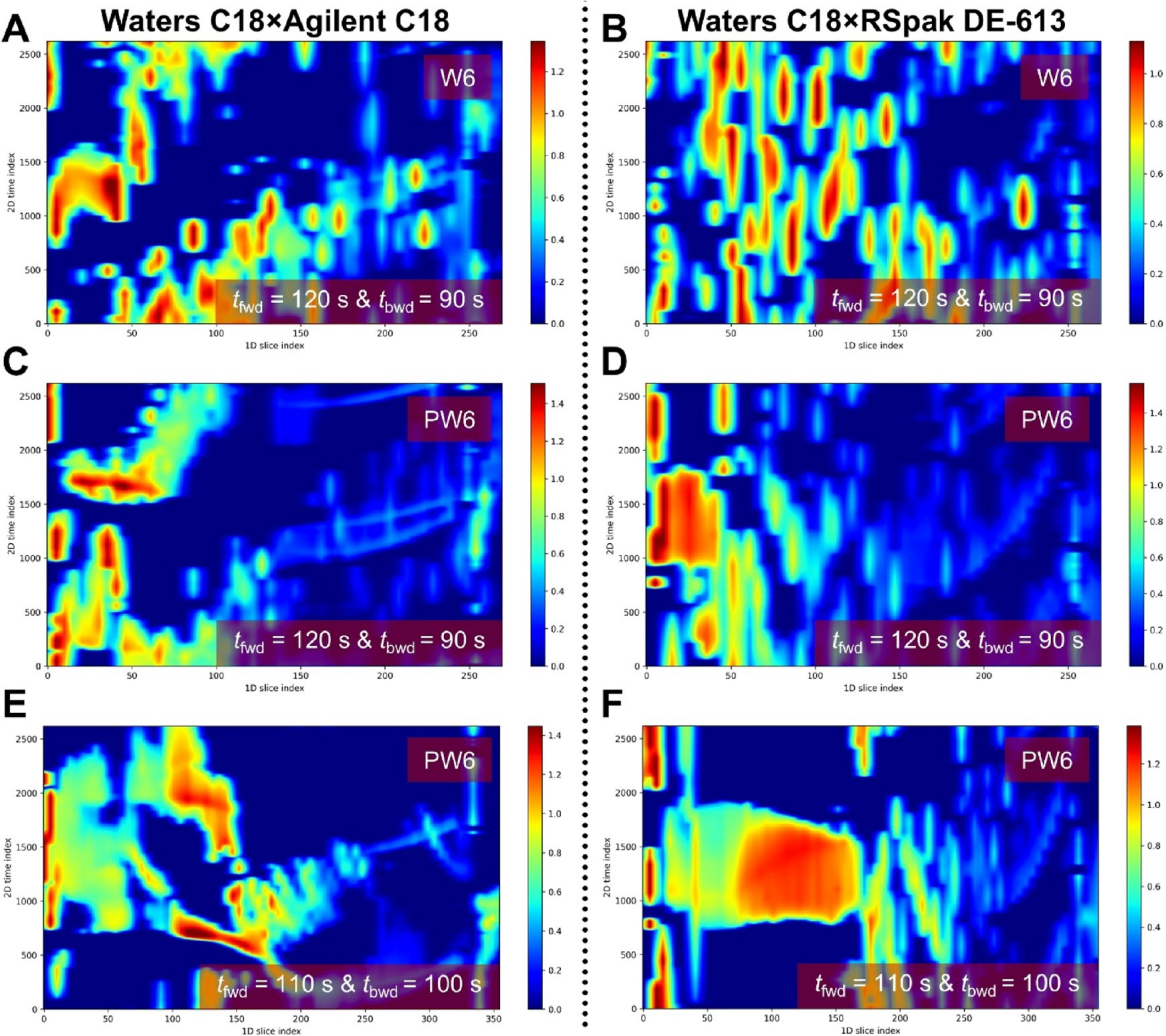
2D control plots using ^1^D Waters C18 with different ^2^D-column and *t*_fwd_: (A) ^2^D Agilent C18 and (B) ^2^D RSpak DE-613 for the separation of W6 with *t*_fwd_ = 120 s. The corresponding results for PW6 are shown in parts C and D, respectively. E and F show the PW6 separation results obtained under the same conditions, but with *t*_fwd_ = 110 s.

The effect of sample complexity was further evaluated using preconcentrated W6 (PW6), which contains a wider range of compounds. This is shown in Figure 6C–F, where the chromatographic patterns become more crowded compared with the DCM wine extract (Figure 6A and B). For the ^1^D Waters C18 × ^2^D Agilent C18 system (Figure 6C and E), peaks remain relatively well distributed across the 2D separation space. While Figure 6C was obtained under the same modulation conditions as those of W6 (*t*_fwd_ = 120 s and *t*_bwd_ = 90 s), the modulation times were adjusted in Figure 6E (*t*_fwd_ = 110 s and *t*_bwd_ = 100 s) to improve fraction transfer and reduce potential peak overlap. In contrast, the ^1^D Waters C18 × ^2^D RSpak DE-613 system (Figure 6D and F) exhibits a more pronounced clustering of peaks, particularly in the early ^1^D fractions, along with increased wraparound contributions in the later-eluting ^1^D fractions.

### Performance and Sustainability Assessment of FBF-VAST Modulation

Apart from its analytical application, the operational performance and sustainability of the proposed FBF-VAST configuration were benchmarked against those of other LC × LC modulation strategies (Table S5). The FBF-VAST system requires only a single pump and one four-port valve, representing the simplest gradient-compatible design capable of forward–backward switching and selectivity tuning while maintaining backflush functionality. Compared with passive modulation, FBF-VAST generates narrower transfer pulses and improved analyte focusing (0.5–3.0× enrichment) through solvent-assisted accumulation during the forward–backward cycles. Although its focusing efficiency remains below that of active or cryogenic/thermal modulators, FBF-VAST markedly reduces hardware cost, solvent and energy use, and maintenance demands.

It is noteworthy that both the single-pump FBF-VAST and passive modulators consume a similar total mobile phase volume at 0.5 mL min^–1^ (≈90 mL per 3 h run), yet their effective solvent utilization differs substantially. In FBF-VAST, optimized flow reversal and minimal purge requirements reduce discarded solvent to ≈8–23 mL per run, compared with ≈30–70 mL for a conventional passive setup. This efficiency gain stems from precise pulse control without the need for additional pump heads or long equilibration cycles. Consequently, FBF-VAST also operates at a lower average power (≈20 W) than typical passive systems (≈25–30 W), which often demand auxiliary actuation or higher pump head work.

By contrast, active dual-pump and cryogenic/thermal modulators, while capable of intense analyte focusing (5–100× enrichment), exhibit substantially higher solvent consumption (150–300 mL h^–1^ and >300 mL h^–1^, respectively) and power demand (>200 W) due to continuous re-equilibration and refrigeration. Cryogenic/thermal designs also require separate cooling loops and cannot operate with a single pump. Overall, the FBF-VAST system achieves one of the highest sustainability ratings (green-chemistry score = 9–10/12) and the lowest life-cycle impact, offering a balanced compromise between chromatographic performance, simplicity, and environmental responsibility. This makes it a practical, retrofittable, and cost-effective LC × LC solution for complex metabolomic and compositional studies such as wine analysis.

## Conclusions

The FBF-VAST (Forward–Backward-Flushing Valve-Assisted Selectivity Tuning) mechanism introduces a simplified yet highly tunable modulation strategy for LC × LC. By alternating flow directions through a single-pump, single-valve setup, it enables analyte retention, release, and gradient compatibility within one continuous operation. Fine selectivity control is achieved by adjusting the forward/backward flushing durations, eliminating the need for additional pumps or complex plumbing.

A key novelty of this approach lies in its built-in backflushing capability, which allows online ^1^D-column regeneration and removal of retained residues without interrupting analysis—enhancing robustness and extending column life. Equally important, the integrated mechanistic simulation visualizes solvent and analyte propagation through each modulation cycle, revealing how *t*_fwd_ and *t*_bwd_ govern selectivity and providing theoretical insight and predictive capability for experimental design.

Experimental results with wine samples confirmed the modeled trends, demonstrating high reproducibility and an effective 2D coverage. With its minimal solvent use, low energy demand, and self-cleaning capability, FBF-VAST offers a sustainable, flexible, and mechanistically guided platform for next-generation multidimensional liquid chromatography.

## Supplementary Material











## Notes

Description of wine samples, stop-flow configuration, gradient mobile phase system, LC × LC chromatograms, and qualitative and quantitative analysis results of the wine samples (PDF)
Summary of experimental conditions and rationale (XLSX)
Dynamic evolution of the mobile phase profile (GIF)
Video S1: Analyte movement and detector signal of resveratrol (MP4)
Video S2: Analyte movement and detector signal of all analytes (MP4)
