# Supplementary figures and images for: Forward–Backward-Flushing Valve-Assisted Selectivity Tuning (FBF-VAST) for LC × LC: Principles and Demonstration of a Modulation Mechanism
Source: Anal Chem. 2026 May 11;98(20):14858–72. doi: 10.1021/acs.analchem.5c08280 (PMC13217371; doi:10.1021/acs.analchem.5c08280)

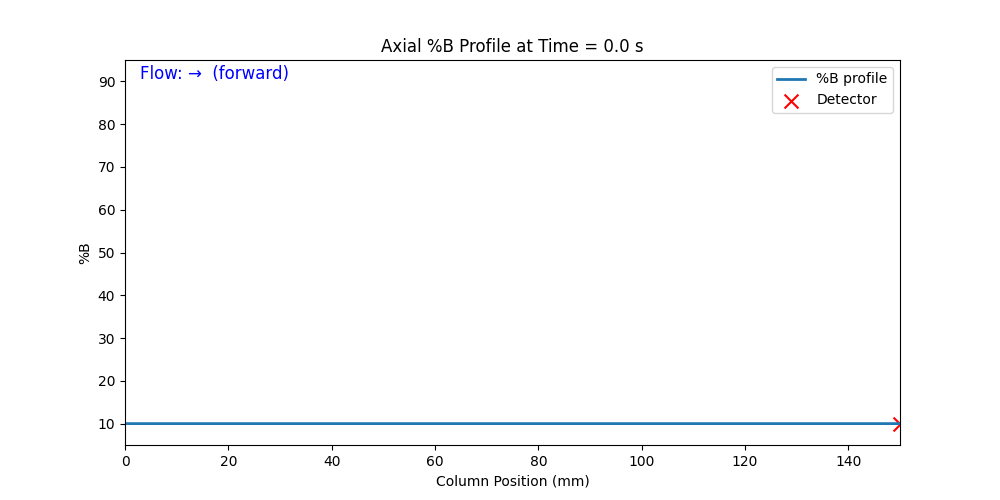

Supplement: Supplementary file 1 [file ac5c08280_si_001.gif]
