# Supplementary material for: Forward–Backward-Flushing Valve-Assisted Selectivity Tuning (FBF-VAST) for LC × LC: Principles and Demonstration of a Modulation Mechanism
Source: Anal Chem. 2026 May 11;98(20):14858–72. doi: 10.1021/acs.analchem.5c08280 (PMC13217371; doi:10.1021/acs.analchem.5c08280)
Supplement: Supplementary file 3 [file ac5c08280_si_003.pdf]

Supporting information

Forward-Backward -Flushing Valve-Assisted Selectivity Tuning (FBF-VAST) for LC×LC: Principles and Demonstration of a Modulation Mechanism

*Pattraporn Chobpradit[1], Kenji Hamase[2], Sornkanok Vimolmangkang[3,4] Thumnoon Nhujak[1] and Chadin Kulsing[1,5]**

[1]Department of Chemistry, Faculty of Science, Chulalongkorn University, Bangkok 10330, Thailand

[2]Graduate School of Pharmaceutical Sciences, Kyushu University, 3-1-1 Maidashi, Higashi-ku, Fukuoka 812-8582, Japan

[3]Department of Pharmacognosy and Pharmaceutical Botany, Faculty of Pharmaceutical Sciences, Chulalongkorn University, Bangkok 10330, Thailand

[4]Phyto Analytica Testing Laboratory, Leapdelab Company Limited, Samut Prakan 10130, Thailand

[5]Center of Excellence in Metabolomics for Life Sciences, Chulalongkorn University, Bangkok 10330, Thailand

*Chadin Kulsing: ckulsing@gmail.com; Tel: +6680291178

TABLE OF CONTENTS

Page

Table S1. Wine samples code, name, type, origin and number of separated peaks. S4

Table S2. The details of each gradient mobile phase system S5

Table S3. Repeatability results of 5 peaks sextuplicate analysis from peak area.................S10

Table S4. Qualitative and quantitative analysis of 10 std in 10 wine samples and linear equation of each standard. S11

Table S5. Comparative assessment of key operational, environmental, and economic parameters among different LC×LC modulation strategies. The developed FBF-VAST configuration operates with a single pump and one 4-port valve, offering full gradient compatibility with significantly reduced solvent and energy consumption while maintaining efficient modulation performance. S12

FIGURE OF CONTENTS

Page

Figure S1. Schematic diagram of stop-flow LCxLC configuration. S4

Figure S2. Comparison of chromatographic peak profiles for three standards obtained using conventional 1D-LC (top) and the proposed FBF-VAST LC×LC system (bottom). S6

Figure S3. Simulated resveratrol peak profiles (above) and corresponding detector signals (below) under FBF-VAST modulation, using only the ^{1}D Waters C18 column. Results are resveratrol at different times following injection (A-H, respectively)...................................S7

Figure S4. Analyte migration overlay map of gallic acid (red), caffeine (green), resveratrol (blue), coumaric acid (yellow), and benzoic acid (purple). S7

Figure S5. HPLC chromatograms of FBF-VAST LC×LC operated under constant valve A position (A) and constant valve B position (B). S8

Figure S6. The comparison of 2D-control plot of FBF-VAST (A) and stop-flow (B) LCxLC in GMP3...S8

Figure S7. Comparison of back pressure profile...S9

Figure S8. Example UV spectra of target peaks for repeatability evaluation of FBF-VAST LC×LC, obtained in scan mode using the UV–Vis detector...S9

Table S1. Wine samples code, name, type, origin and number of separated peaks.

Code	Name	Fruit type	Location
W1	Cape barren McLAREN vale 2015	Shiraz	McLaren Vale, Australia
W2	Fat bastard syrah 2016	Shiraz	Béziers, France
W3	PB Khao yai reserve shiraz 2012	Shiraz	Nakhonratchasima, Thailand
W4	PB Khao yai reserve shiraz 2012	Shiraz	Nakhonratchasima, Thailand
W5	Chateau De Loei 2013	Shiraz	Loei, Thailand
W6	Silverlake 2014	Shiraz	Chonburi, Thailand
W7	Silverlake 2012	Shiraz	Chonburi, Thailand
W8	Fritz salomon gut oberstockstall 2014	Riesling	Wagram, Austria
W9	Knight black house 2015	Fruit (Lychee)	Phathum-Thani, Thailand
W10	Mythical garden 2017	Fruit (Mangosteen)	Phathum-Thani, Thailand

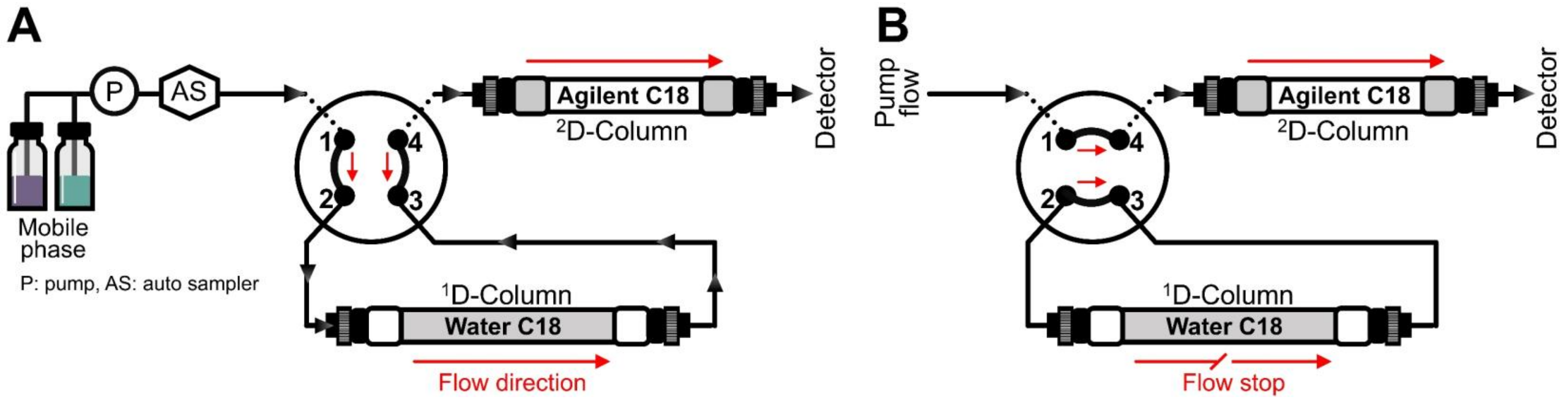

Figure S1. Schematic diagram of stop-flow LCxLC configuration.

Table S2. The details of each gradient mobile phase system

Gradient	Time (min)	%H_2O	%ACN	Description	Number of peaks*
**GMP1**	0.00	90.00	10.00	initial	18
	5.00	90.00	10.00	pre-equilibration	
	20.00	10.00	90.00	linear gradient	
	100.00	10.00	90.00	wash	
	100.10	90.00	10.00	return to initial	
	120.00	90.00	10.00	equilibrate	
**GMP2**	0.00	90.00	10.00	initial	34
	5.00	90.00	10.00	pre-equilibration	
	80.00	10.00	90.00	linear gradient	
	100.00	10.00	90.00	wash	
	100.10	90.00	10.00	return to initial	
	120.00	90.00	10.00	equilibrate	
**GMP3**	0.00	90.00	10.00	initial	46
	5.00	90.00	10.00	pre-equilibration	
	120.00	10.00	90.00	linear gradient	
	130.00	10.00	90.00	wash	
	130.10	90.00	10.00	return to initial	
	150.00	90.00	10.00	equilibrate	
**GMP4**	0.00	90.00	10.00	initial	57
	5.00	90.00	10.00	pre-equilibration	
	160.00	10.00	90.00	linear gradient	
	170.00	10.00	90.00	wash	
	170.10	90.00	10.00	return to initial	
	190.00	90.00	10.00	equilibrate	
**GMP5**	0.00	90.00	10.00	initial	64
	5.00	90.00	10.00	pre-equilibration	
	230.00	10.00	90.00	linear gradient	
	240.00	10.00	90.00	wash	
	240.10	90.00	10.00	return to initial	
	250.00	90.00	10.00	equilibrate	
**GMP6**	0.00	90.00	5.00	initial	
	65.00	90.00	10.00	pre-equilibration	
	230.00	10.00	90.00	linear gradient	
	240.00	10.00	90.00	wash	
	240.10	90.00	5.00	return to initial	
	250.00	90.00	5.00	equilibrate	

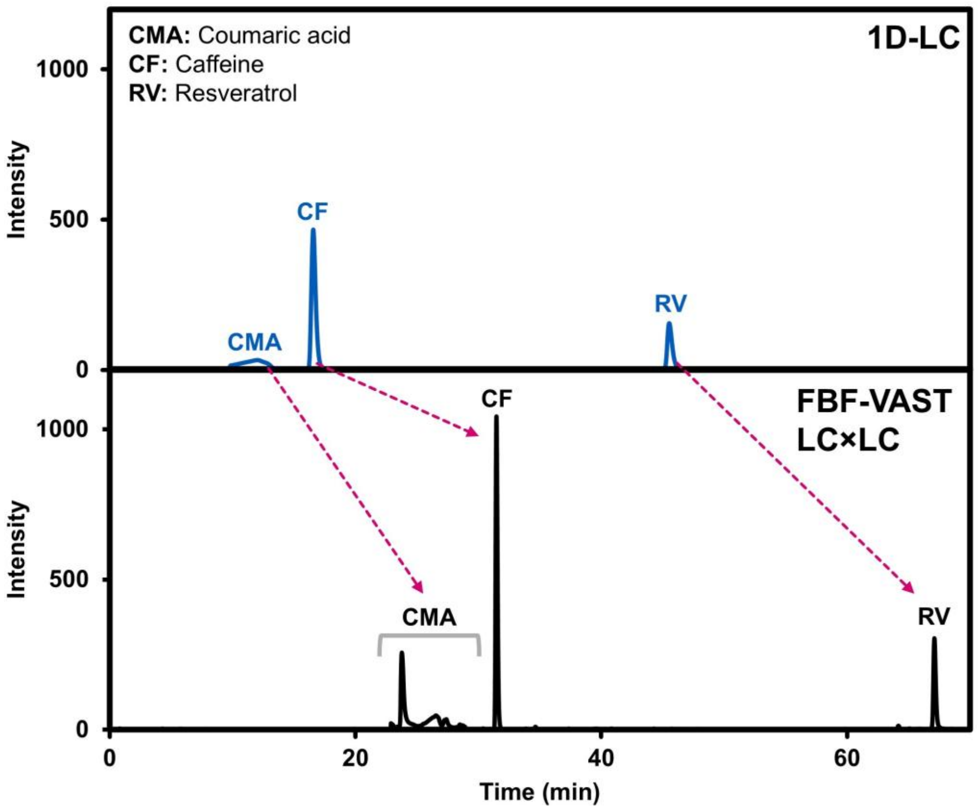

Figure S2. Comparison of chromatographic peak profiles for three standards obtained using conventional 1D-LC (top) and the proposed FBF-VAST LC×LC system (bottom).

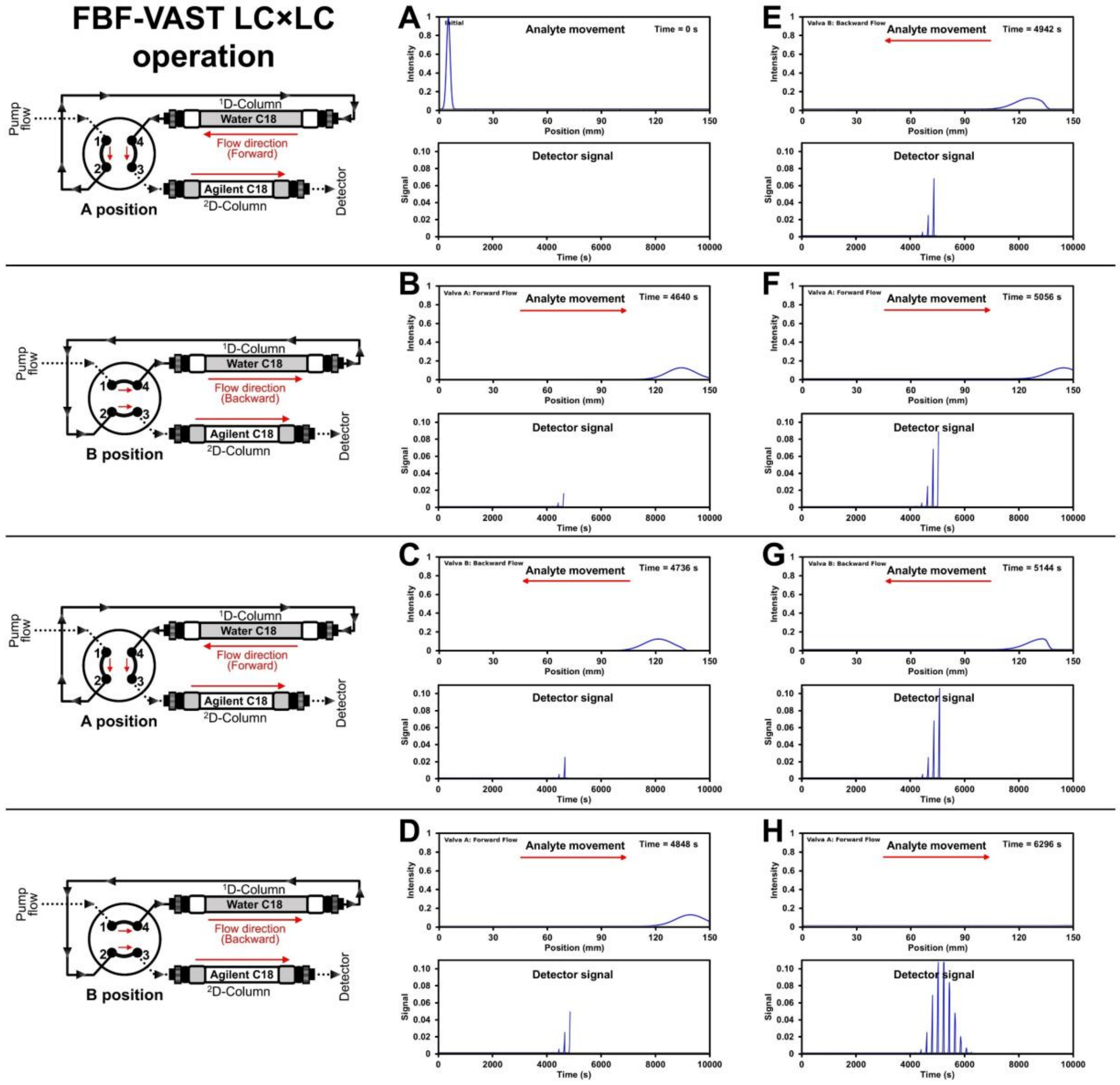

Figure S3. Simulated resveratrol peak profiles (above) and corresponding detector signals (below) under FBF-VAST modulation, using only the [1]D Waters C18 column. Results are resveratrol at different times following injection (A-H, respectively).

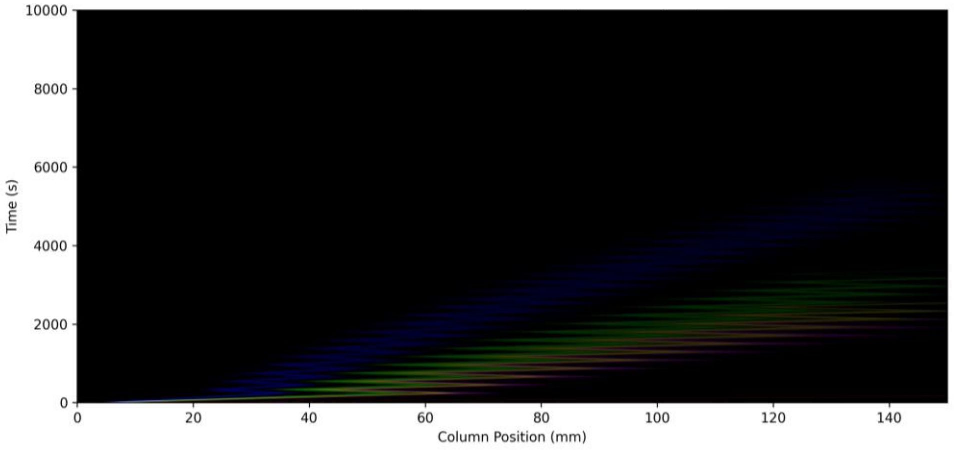

Figure S4. Analyte migration overlay map of gallic acid (red), caffeine (green), resveratrol (blue), coumaric acid (yellow), and benzoic acid (purple).

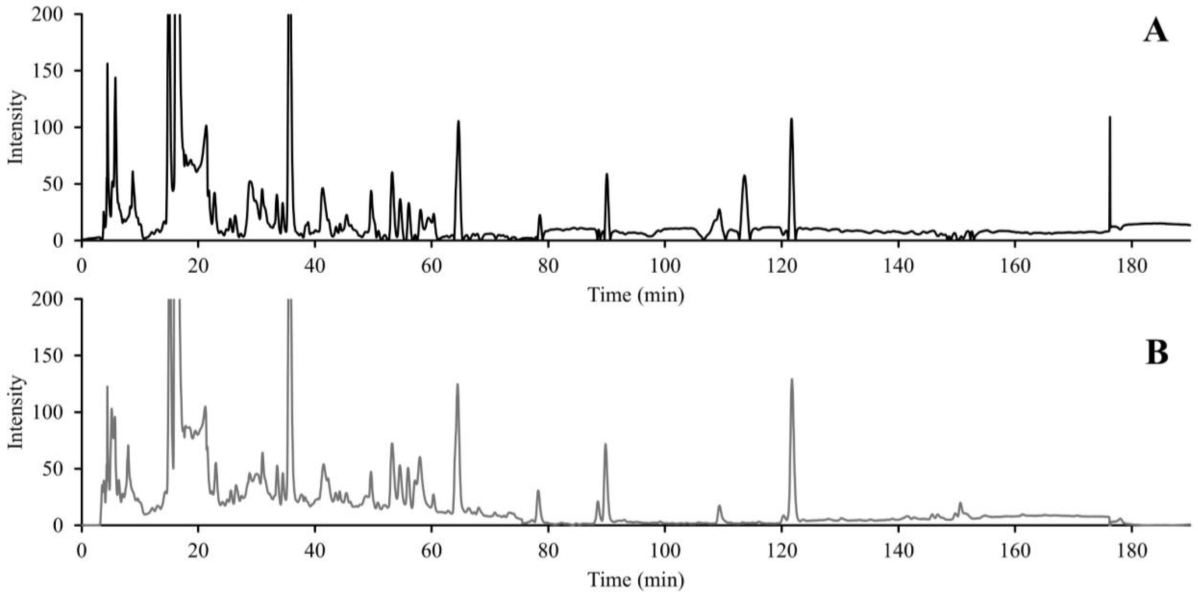

Figure S5. HPLC chromatograms of FBF-VAST LC×LC operated under constant valve A position (A) and constant valve B position (B).

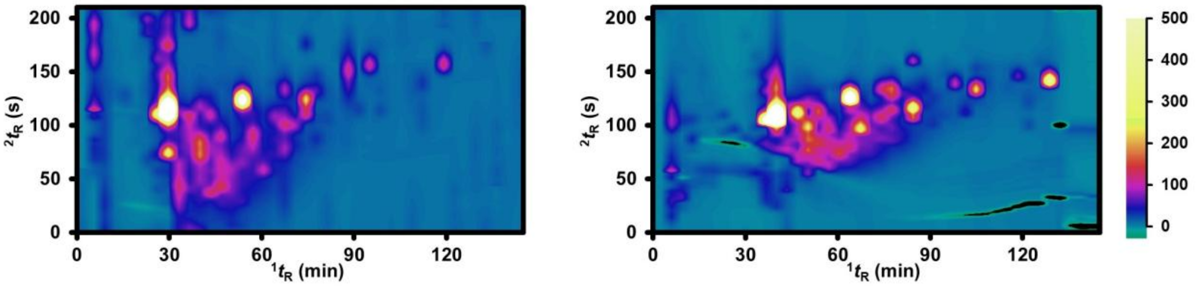

Figure S6. The comparison of 2D-control plot of FBF-VAST (A) and stop-flow (B) LCxLC under GMP3.

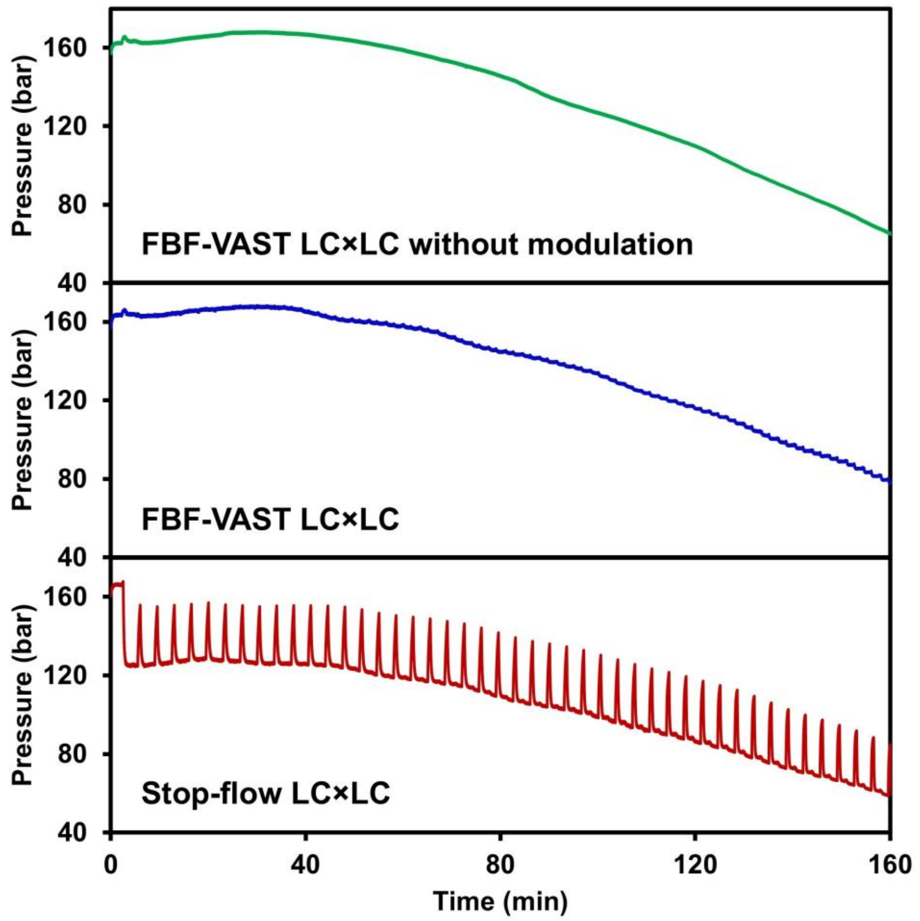

Figure S7. Comparison of back pressure profiles.

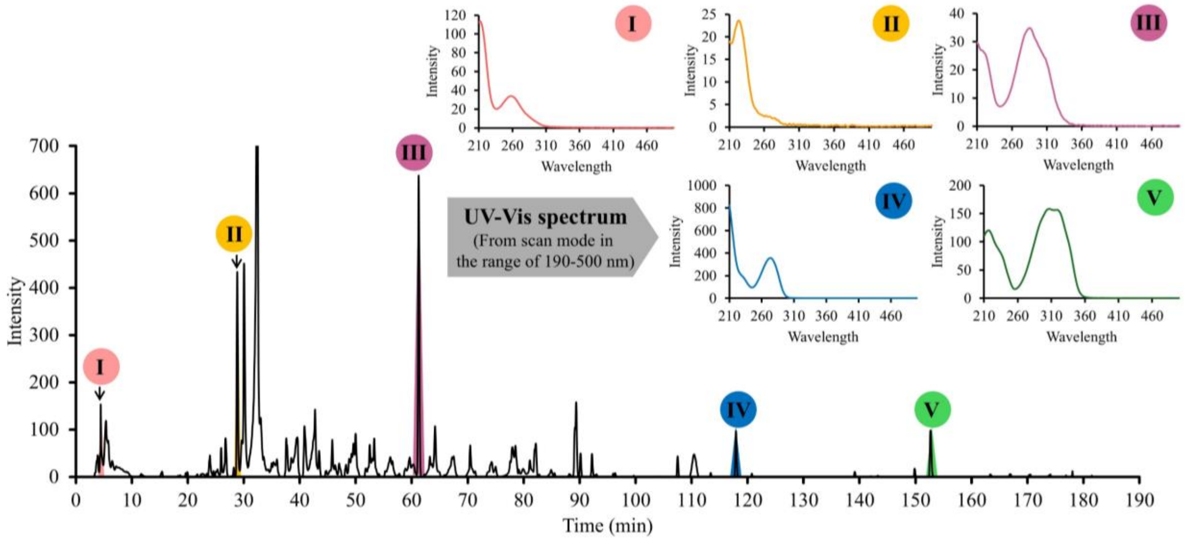

Figure S8. Example UV spectra of target peaks for repeatability evaluation of FBF-VAST LC×LC, obtained in scan mode using the UV–Vis detector.

Table S3. Repeatability results of 5 peaks sextuplicate analysis from peak area.

Peak no.	FBF-VAST (%RSD)	Stop-flow (%RSD)
1	8.14	10.43
2	7.66	11.35
3	5.28	9.59
4	6.91	5.53
5	11.77	11.84

Table S4. Qualitative and quantitative analysis of 10 target compounds in 10 wine samples and linear equation of each compound.

Standard compound	Linear equation	R^2	Concentration of target compounds (mg/g of wine)									
			Red wine							White wine	Fruit wine	
			W1	W2	W3	W4	W5	W6	W7	W8	W9	W10
Phenolic acids												
Benzoic acid	y = 76.7x - 195.1	0.989	n.d.	n.d.	n.d.	n.d.	n.d.	n.d.	n.d.	n.d.	n.d.	0.016
Caftaric acid	y = 161.9x + 150.8	0.997	n.d.	n.d.	0.002	0.007	0.008	0.002	n.d.	0.022	0.004	n.d.
Chlorogenic acid	y = 62.6x - 455.2	0.967	n.d.	n.d.	n.d.	0.013	0.016	n.d.	n.d.	n.d.	n.d.	n.d.
p-Coumaric acid	y = 275.7x - 381.2	0.989	0.017	0.029	0.045	0.066	0.042	0.037	0.034	0.015	0.008	n.d.
Vanillic acid	y = 155.7x + 85.3	0.995	n.d.	n.d.	0.001	0.003	n.d.	0.003	0.001	n.d.	n.d.	n.d.
Flavonoids												
(+)-Catechin	y = 161.4x + 138.7	0.984	n.d.	0.024	0.002	0.042	n.d.	0.044	n.d.	n.d.	0.011	n.d.
(-)-Epicatechin	y = 420.7x + 204.2	0.997	n.d.	n.d.	n.d.	0.002	0.006	0.002	0.003	0.002	0.005	0.004
Other												
Caffeine	y = 168.2x - 159.7	0.998	n.d.	n.d.	n.d.	n.d.	n.d.	n.d.	n.d.	n.d.	n.d.	n.d.
Nicotinic acid	y = 69.6x + 37.9	0.996	0.013	0.010	0.011	0.004	0.005	0.010	0.011	n.d.	0.011	0.008
Resveratrol	y = 72.5x - 108.3	0.989	0.009	0.035	0.012	0.039	0.028	0.031	0.022	n.d.	0.010	n.d.

n.d.; non-detectable

Table S5. Comparative assessment of key operational, environmental, and economic parameters among different LC×LC modulation strategies. The developed FBF-VAST configuration operates with a single pump and one 4-port valve, offering full gradient compatibility with significantly reduced solvent and energy consumption while maintaining efficient modulation performance.

Parameter	FBF-VAST (This Work)	Passive Modulation	Active Modulation (Dual Pump)	Cryogenic / Thermal Modulation
**No. of Pumps**	1	1–2	2	1–2 + coolant system
**No. of Valves**	1 × 4-port	2 × 6- or 10-port	≥ 2 × 6/10-port	≥ 2 × 10-port + T-unions
**Ease of Retrofitting**	Excellent	Good	Difficult	Very difficult
**Hardware Cost (USD)**	~ 5,000–10,000	~ 10,000–20,000	~ 18,000–30,000	~ 50,000–80,000 +
**Transfer Pulse Width (Δt_{net})**[a]	~ 5–90 s	Broad	~ 2–10 s	~ 1–3 s
**Analyte Enrichment factor**[b]	0.5–3×	1× (none)	5–20×	10–100×
**Gradient Compatibility**[c]	Yes	Limited	Yes	No
**Solvent Use (mL/h)**[d]	~ 25–35 mL/h	~ 35–80 mL/h	~ 40–90 mL/h	> 100 mL/h
**Waste Volume (per run)**[e]	~ 8–23 mL (3 h run)	~ 30–70 mL (3 h run)	~ 40–85 mL (3 h run)	Very high (> 150 mL)
**Energy Use (W/run)**[f]	~ 10–20 W/run	~ 15–30 W/run	~ 30–50 W/run	> 200 W/run
**Instrumental Footprint**	Very compact	Moderate	Large	Very large
**Maintenance (annually)**[g]	Low–Moderate	Low–Moderate	Moderate–High	Very high
**Green Chemistry Score**[h]	9–10	6–8	6–7	2–4
**ROI (Payback Period)**	< 6 months	~ 1–2 years	~ 2–4 years	> 5 years
**LCA Score (Impact Units)**[i]	Low (1–2)	Moderate (3–5)	High (6–8)	Very high (> 9)

[a] **Transfer pulse width (Δt_{net}):**
Effective duration of analyte transfer from the first to the second dimension, accounting for valve actuation delay, loop volume, and flow rate. Narrower Δt_{net} corresponds to better focusing and higher modulation efficiency.

[b] **Analyte focusing/enrichment factor:**
Ratio of peak height (or area concentration) obtained after modulation to that before modulation, reflecting solvent-based analyte accumulation or compression efficiency during modulation.

[c] **Gradient compatibility:**
Indicates whether the modulation approach allows use of independent solvent gradients in both dimensions without solvent-strength mismatch. “Limited” means partial compatibility or reduced reproducibility under gradient conditions.

[d] **Solvent use (mL/h):**
Total mobile phase volume pumped from the reservoirs during the entire run, including analytical eluent, gradient blending, solvent-assist pulses, washing, and column conditioning steps. Calculated as total flow rate × h, plus auxiliary flush volumes.

[e] **Waste volume (mL/run):**
Total solvent volume discarded during operation (e.g., equilibration discards, loop purges, and conditioning flushes). Analytical effluent collected for detection or fractionation (e.g., MS, UV, or sample collection) is not counted as “waste.”

[f] **Energy use (W/run):**
Estimated from the electrical power draw of the primary pump(s), valve actuators, and auxiliary components, multiplied by run duration.
Typical values were measured or approximated from manufacturer specifications and representative laboratory power meters.

[g] **Maintenance (annual):**
Approximate routine service and consumable costs, including valve seals, tubing, solvent filters, and coolant maintenance (for thermal/cryogenic systems).

[h] **Green Chemistry Score (0–12):**
A semi-quantitative sustainability index based on twelve weighted criteria including solvent use, waste generation, energy consumption, recyclability, maintenance demand, and safety considerations. Higher scores indicate greener operation.

[i] **ROI (Return on Investment):**
Estimated payback period based on the hardware cost offset by solvent, energy, and maintenance savings compared to a conventional dual-pump active LC×LC configuration.

[j] **LCA (Life Cycle Assessment) Score:**
Relative environmental impact index (arbitrary scale 1–10) integrating cumulative solvent and energy use, hardware mass, maintenance requirements, and waste management burden. Lower values correspond to lower life-cycle impact.
